# Midbrain dopamine oxidation links ubiquitination of glutathione peroxidase 4 to ferroptosis of dopaminergic neurons

**DOI:** 10.1172/JCI165228

**Published:** 2023-05-15

**Authors:** Jie Sun, Xiao-Min Lin, Dan-Hua Lu, Meng Wang, Kun Li, Sheng-Rong Li, Zheng-Qiu Li, Cheng-Jun Zhu, Zhi-Min Zhang, Chang-Yu Yan, Ming-Hai Pan, Hai-Biao Gong, Jing-Cheng Feng, Yun-Feng Cao, Feng Huang, Wan-Yang Sun, Hiroshi Kurihara, Yi-Fang Li, Wen-Jun Duan, Gen-Long Jiao, Li Zhang, Rong-Rong He

**Affiliations:** 1 The First Affiliated Hospital of Jinan University, Guangdong Engineering Research Center of Chinese Medicine & Disease Susceptibility, International Cooperative Laboratory of Traditional Chinese Medicine Modernization and Innovative Drug Development of the Chinese Ministry of Education, Guangdong Province Key Laboratory of Pharmacodynamic Constituents of Traditional Chinese Medicine and New Drugs Research, and The Sixth Affiliated Hospital of Jinan University, Jinan University, Guangzhou, China.; 2 Shanghai Institute for Biomedical and Pharmaceutical Technologies, National Health Commission Key Laboratory of Reproduction Regulation, Shanghai, China.; 3 School of Chinese Materia Medica and Yunnan Key Laboratory of Southern Medicinal Utilization, Yunnan University of Chinese Medicine, Kunming, China.; 4 Key Laboratory of CNS Regeneration, Ministry of Education, Guangdong-Hong Kong-Macau Institute of CNS Regeneration, Jinan University, Guangzhou, China.; 5 State Key Laboratory of Quality Research in Chinese Medicine, Macau University of Science and Technology, Macau, China.

**Keywords:** Cell Biology, Neuroscience, Parkinson disease, Ubiquitin-proteosome system

## Abstract

Parkinson’s disease (PD) is a neurodegenerative disorder characterized by the gradual loss of midbrain dopaminergic neurons in association with aggregation of α-synuclein. Oxidative damage has been widely implicated in this disease, though the mechanisms involved remain elusive. Here, we demonstrated that preferential accumulation of peroxidized phospholipids and loss of the antioxidant enzyme glutathione peroxidase 4 (GPX4) were responsible for vulnerability of midbrain dopaminergic neurons and progressive motor dysfunctions in a mouse model of PD. We also established a mechanism wherein iron-induced dopamine oxidation modified GPX4, thereby rendering it amenable to degradation via the ubiquitin-proteasome pathway. In conclusion, this study unraveled what we believe to be a novel pathway for dopaminergic neuron degeneration during PD pathogenesis, driven by dopamine-induced loss of antioxidant GPX4 activity.

## Graphical abstract

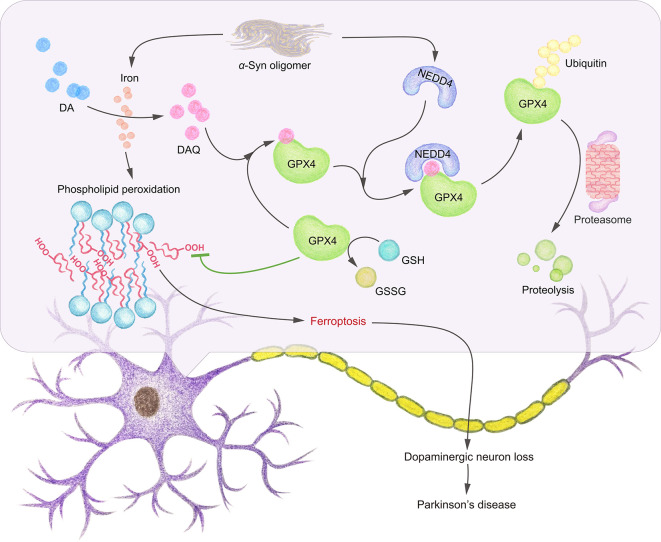

## Introduction

Parkinson’s disease (PD) is a neurodegenerative disorder characterized by motor symptoms, including tremor, rigidity, and abnormal gait. These are collectively referred to as parkinsonism (1), and they are the result of the progressive loss of dopaminergic neurons in substantia nigra (2) along with the formation of Lewy bodies that comprise misfolded α-synuclein in the neurons (3). Interestingly, PD is also associated with iron overload and lipid peroxidation, which are highly consistent with ferroptosis, a form of regulated cell death that is initiated by iron-dependent phospholipid peroxidation (4). We previously reported that ferroptotic cell death is a potential mechanism for loss of dopaminergic neurons (5). Indeed, iron-dependent neuron loss was described in α-synuclein–induced neuronal death well before the emerging concept of ferroptosis (6), though evidence linking lipid peroxidation, ferroptosis, and synucleinopathy has been lacking.

Dopaminergic neurons are the core factories responsible for producing dopamine (DA), an unstable neurotransmitter that can cause oxidative toxicity in the iron-rich substantia nigra environment. Labile iron accelerates the spontaneous oxidation of DA to an electron-deficient DA quinone (DAQ), which becomes covalently bound to nucleophiles, such as the thiol group in the amino acid cysteine (7). Glutathione peroxidase 4 (GPX4), an antioxidant protein that is highly expressed in the brain, has been suggested as a likely target of DAQ modification (8). It is worth noting that GPX4 is the only known mammalian enzyme that can directly catalyze the reduction of hydroperoxides in phospholipids to effectively suppress ferroptosis (9). Inhibition of GPX4 or its upstream factors can induce ferroptosis; for instance, blockade of GPX4 with RSL3 or System X_c_^–^ with sorafenib (10, 11). Therefore, we hypothesized that irreversible covalent modification of GPX4 by DAQ may alter its function, and, subsequently, activate the ferroptotic pathway, to modulate dopaminergic neuron degeneration.

The A53T mutation in the α-synuclein gene (*SNCA*^A53T^, or A53T) is one of the key missense gene mutations linked to early onset familial PD, as the mutated α-synuclein variant is prone to self-aggregation (12). Additionally, the mutated variant disrupts the physiological ferrireductase activity and leads to dysregulation of iron metabolism (13–15) and membrane lipid oxidation (5). It has been previously speculated that α-synuclein oligomers exert neurotoxicity by directly disrupting the lipid bilayer in the plasma membrane (16). However, murine A53T mutations displayed normal membrane-binding activity (17). This raises the possibility of other players contributing to the etiology of PD. In this project, we investigated the link between dopamine, GPX4, and lipid peroxidation, and their relevance to PD.

## Results

### Phospholipid peroxidation leads to dopaminergic neuron loss in synucleinopathies.

**Figure 1:**
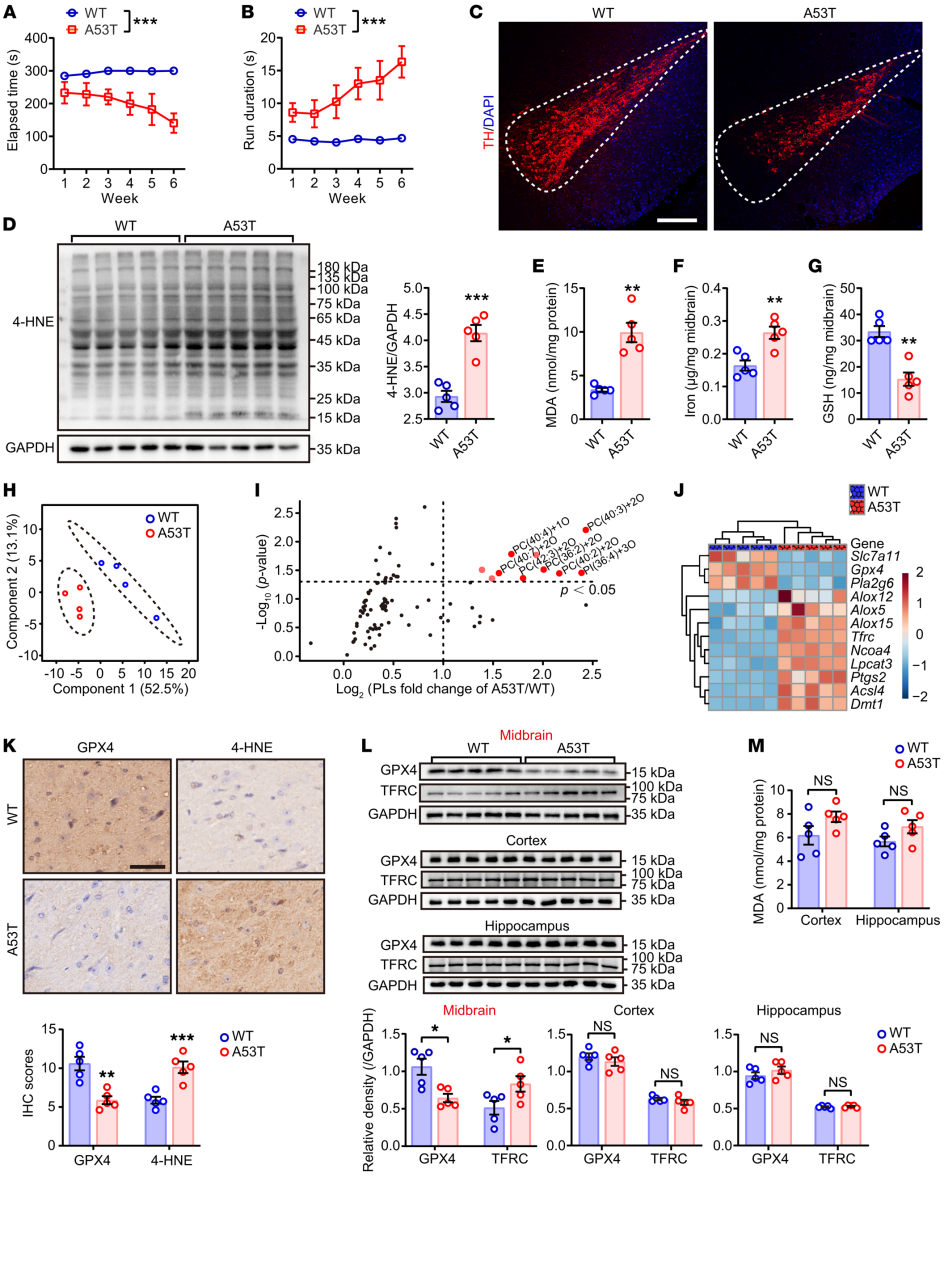
Ferroptosis pathway is involved in PD progression in *SNCA*^A53T^ transgene mice. Disordered motor coordination was evaluated in 13-month-old *SNCA*^A53T^ mice using the rotarod test (**A**, the time latency to drop from the rotarod) and pole climbing (**B**, the time latency to reach the bottom of the pole). WT mice were used as controls. Significance was determined by 2-way repeated measures ANOVA (*n* = 5). (**C**) Immunofluorescence of substantia nigra sections (dotted area) labeled with TH antibody (red) and DAPI (blue). Scale bar: 200 μm. (**D**) Western blotting (left) and quantitative analysis (right) of 4-HNE expression in midbrain. (**E**) MDA, (**F**) iron, and (**G**) glutathione (GSH) in midbrain were measured (*n* = 5). The phospholipids in midbrain were isolated and detected by LC-MS/MS (*n* = 4). Data of phospholipids were extracted and displayed as (**H**) principal component analysis (PCA) and (**I**) volcano plots showing the fold changes (X-axis) versus significance (Y-axis). (**J**) Ferroptosis-related genes were detected by qPCR assay and relative expressions were displayed as a heatmap. *Slc7a11*, solute carrier family 7 member 11; *Gpx4*, glutathione peroxidase 4; *Pla2g6*, phospholipase A2 group VI; *Alox5/12/15*, arachidonate 5/12/15-lipoxygenase; *Tfrc*, transferrin receptor; *Ncoa4*, nuclear receptor coactivator 4; *Lpcat3*, lyso-PC acyltransferase 3; *Ptgs2*, prostaglandin-endoperoxide synthase 2; *Acsl4*, acyl-CoA synthetase long chain family member 4; *Dmt1*, ferrous ion membrane transport protein DMT1. (**K**) IHC of midbrain sections labeled with GPX4 and 4-HNE antibodies and hematoxylin. IHC magnification (top) and IHC score (bottom). Scale bar: 50 μm. (**L**) Western blotting (top) and quantitative analysis (bottom) of GPX4 and TFRC expression in midbrain, cortex, and hippocampus, respectively. (**M**) MDA level was measured in the cortex and hippocampus, respectively (*n* = 5). All data represent mean ± SEM. *P < 0.05, **P < 0.01 and ***P < 0.001, by independent-samples *t*-test.

Synucleinopathy is believed to be responsible for parkinsonism. Transgenic mice overexpressing *SNCA*^A53T^ displayed progressively deteriorated motor coordination functions in rotarod and pole climbing tests (Figure 1, A and B) and additionally displayed arrhythmic walking patterns (Supplemental Figure 1, A–I; supplemental material available online with this article; https://doi.org/10.1172/JCI165228DS1 Such behavioral deficits in A53T mice were accompanied by the presence of elevated α-synuclein monomers and aggregates (Supplemental Figure 1J) and a remarkable loss of dopaminergic neurons in the substantia nigra, as indicated by tyrosine hydroxylase (TH) staining (Figure 1C). In addition, reduced DA production and elevated inflammatory markers in the substantia nigra compacta and impaired DA metabolism in the striatum were also observed (Supplemental Figure 1, M–Q). These anomalies were associated with significant increases in 4-hydroxynonenal (4-HNE), malondialdehyde (MDA), and iron in the midbrain of the A53T mice (Figure 1, D–F). In contrast, glutathione (GSH) levels were significantly decreased in the midbrain (Figure 1G). Phospholipidomics analysis in midbrain samples from A53T mice revealed distinct patterns of oxidized phospholipids when compared with the WT littermates (Figure 1H). The major species of oxidized phospholipids was identified to be oxidized phosphatidylcholine (PC) (Figure 1I and Supplemental Figure 1R). Taken together, these data indicated a direct correlation between lipid peroxidation and neurodegeneration, with the oxidized phospholipids suggestive of ferroptosis.

Since peroxides of polyunsaturated fatty acid phospholipids (PUFA-PLs) are known to cause ferroptosis (10), we studied the expression of ferroptosis-related genes and proteins. Quantitative real-time PCR analysis revealed an increase in ferroptosis-enhancing genes and a decrease in ferroptosis-inhibiting genes (Figure 1J). Of these, the reduction in GPX4 and elevation of transferrin receptor 1 (TFRC) levels (Figure 1, K and L) were particularly significant since they are characteristic of ferroptotic cell death. Interestingly, 4-HNE accumulation and loss of GPX4 occurred selectively in midbrain nuclei, which suggested that ferroptosis occurred selectively in the midbrain region but not in the cortex and hippocampus (Figure 1K and Supplemental Figure 1, K and L). In contrast, MDA levels and protein expression of GPX4 and TFRC in the cortex and hippocampus of A53T mice were not significantly different from those in WT mice (Figure 1, L and M).

### High α-synuclein increases lipid oxidation, which leads to ferroptosis.

**Figure 2:**
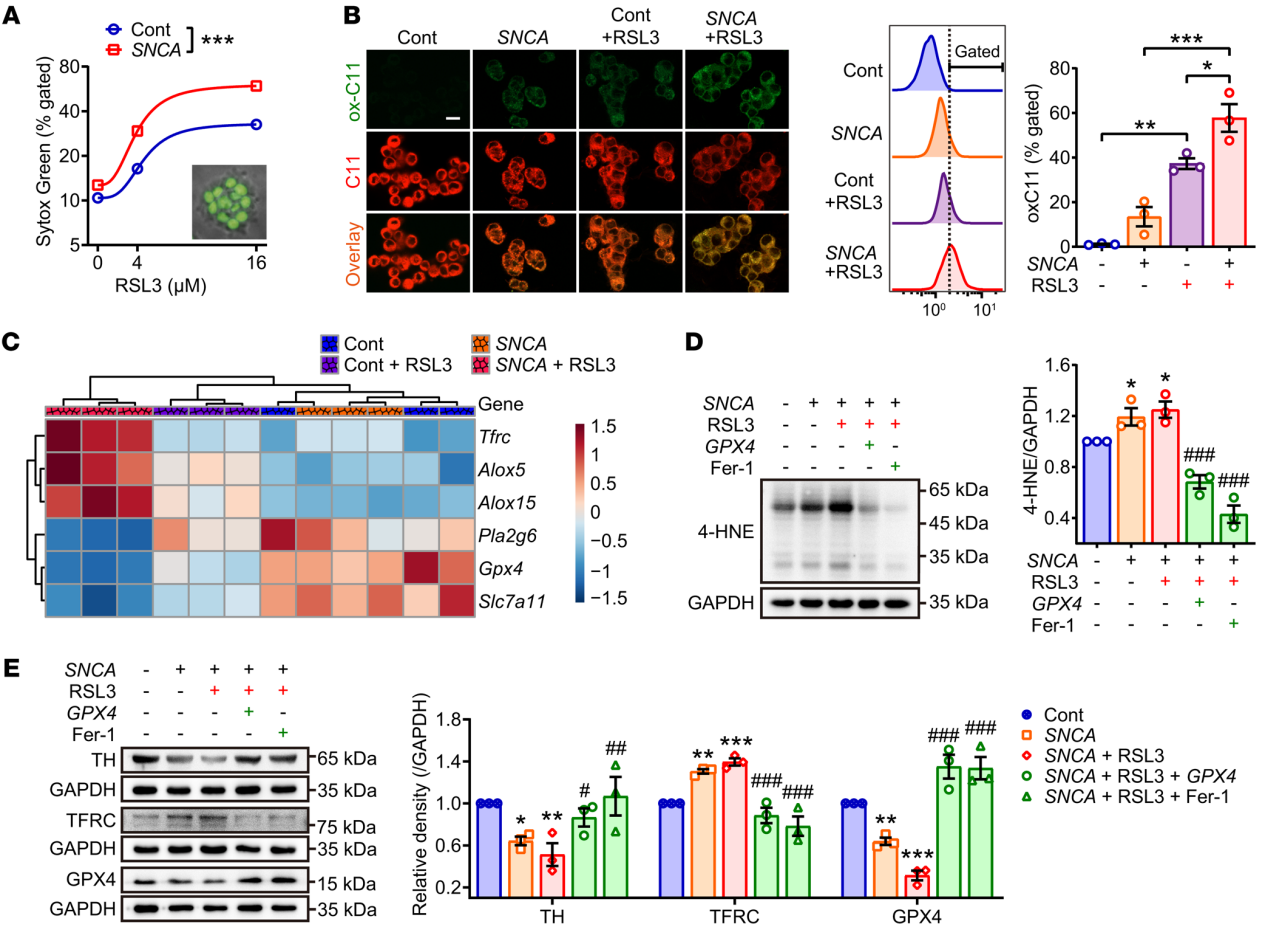
Lipid peroxidation is involved in α-synuclein–induced ferroptotic susceptibility. (**A**) PC12 cell lines carrying doxycycline-inducible *SNCA*^A53T^ were treated with different doses of RSL3 for 12 hours. The cells were incubated with Sytox Green and analyzed by flow cytometry to determine the percentage of cells with compromised membranes (see inset). Data represent mean ± SEM (*n* = 3 independent samples) by 2-way repeated measures ANOVA. (**B**) *SNCA*-overexpressed PC12 cells were treated with RSL3 (12.5 μM) for 6 hours to induce lipid peroxides, which were visualized by confocal laser microscopy (left, ox-C11) and flow cytometry (right). Data were analyzed by 1-way ANOVA with Bonferroni test. (**C**) Representative ferroptosis-related genes were detected by quantitative real-time PCR assay and the relative expression displayed as a heatmap. (**D**) Western blotting (left) and quantitative analysis (right) of 4-HNE expression. (**E**) Western blotting (left) and quantitative analysis (right) of TH, TFRC and GPX4 expressions in PC12 cells treated with indicated agents for 12 hours. RSL3, 12.5 μM. *GPX4*, plasmid expressing GPX4. Fer-1, ferrostatin-1, 10 μM. All data represent mean ± SEM (*n* = 3 independent samples). *P < 0.05, **P < 0.01, and ***P < 0.001 versus the control cells, ^#^*P* < 0.05, ^##^*P* < 0.01 and ^###^*P* < 0.001 versus the RSL3-treated *SNCA*-overexpressed cells, by 1-way ANOVA with LSD posthoc test.

To better quantify the relationship between ferroptosis and synucleinopathy, the PC12 cell line carrying a doxycycline-induced *SNCA*^A53T^ gene construct was tested for 4-HNE and lipid peroxidation after doxycycline induction. As shown in Supplemental Figure 2, A and B, the levels of 4-HNE and lipid ROS in these cells gradually increased with overexpression of α-synuclein. In line with these changes, α-synuclein also increased intracellular iron (Supplemental Figure 2C) and TFRC protein expression and decreased GPX4 protein expression (Supplemental Figure 2D). Furthermore, α-synuclein overexpression compromised cell viability in response to treatment with the ferroptosis inducer RSL3 (Supplemental Figure 2, E and F) in a manner that correlated with an increase in oxidized lipids (Figure 2, A and B). RSL3-mediated oxidative damage was reversed by overexpression of human *GPX4* or the ferroptosis inhibitor ferrostatin-1 (Fer-1), but not by inhibitors of autophagy, necrosis, or apoptosis (Supplemental Figure 2, G and H). In terms of molecular profiling, α-synuclein amplified RSL3, induced perturbation of ferroptosis-related genes (Figure 2C), and promoted accumulation of 4-HNE (Figure 2D) and changes to TH, TFRC, and GPX4 expression (Figure 2E). In short, overexpression of the *SNCA*^A53T^ deteriorated cell health by lipid peroxidation suggestive of ferroptosis.

**Figure 3:**
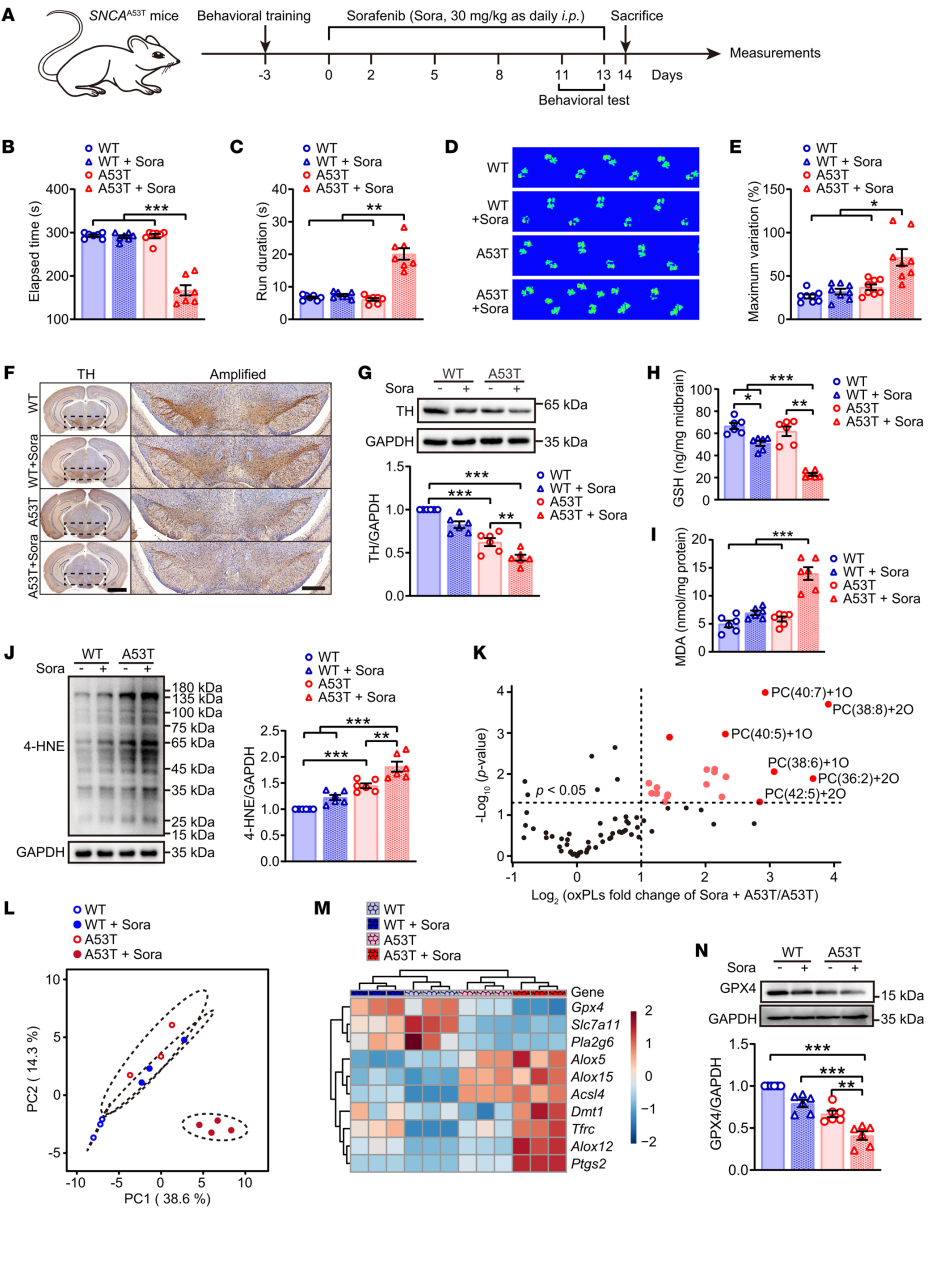
Phospholipid peroxidation is associated with ferroptosis-associated parkinsonism. (**A**) Schematic diagram showing the study design using 8-month-old A53T transgenic mice treated with low-dose sorafenib (Sora) before measurements. Disordered motor coordination of A53T mice was accelerated by Sora, while the WT mice were unaffected, as displayed by behavioral tests (**B**) rotarod, (**C**) pole climbing and (**D** and **E**) Catwalk gait analysis (*n* = 8 mice each group). (**F**) IHC of coronal brain sections labeled with TH antibody and hematoxylin (left, scale bar: 2 mm). The substantia nigra (dotted area) were amplified on right, scale bar: 500 μm. (**G**) Western blotting (top) and quantitative analysis (bottom) of TH expression in midbrain. The contents of (**H**) GSH and (**I**) MDA were measured in midbrain. (**J**) Western blotting (left) and quantitative analysis (right) of 4-HNE expression in midbrain. Data of oxidized phospholipids were extracted and displayed as (**L**) PCA and (**K**) volcano plots showing the fold changes (X-axis) versus significance (Y-axis) by *t*-test. (**M**) Ferroptosis-related genes were detected by quantitative real-time PCR assay and relative expressions displayed as a heatmap. (**N**) Western blotting (upper) and quantitative analysis (bottom) of GPX4 expression in midbrain (*n* = 6 mice each group). All data represent mean ± SEM. *P < 0.05, **P < 0.01 and ***P < 0.001, by 1-way ANOVA with Dunnett T3 (for **B**, **C**, **E**, and **H**) or Bonferroni’s posthoc (for **G**, **I**, **J**, and **N**) test.

To recapitulate α-synuclein–associated lipid oxidative stress in vivo, the ferroptosis inducer sorafenib was administered at a low dosage, around 6 months before the expected onset of overt parkinsonism (Figure 3A). The clinically approved anticancer drug sorafenib has multiple pharmacological effects, such as targeting RAF kinase and the VEGFR-2/PDGFR-β signaling cascade. There is evidence that sorafenib inhibits System X_c_^–^ function and triggers ferroptosis (11), although this effect has been viewed as controversial in several tumor cell lines (18). Behavioral data show that sorafenib significantly increased the vulnerability of A53T mice to parkinsonism, as shown by impaired coordination in rotarod and pole-climbing tests as well as disordered gait patterns (Figure 3, B–E). Sorafenib also aggravated PD-related pathologies such as reduced substantia nigra TH levels (Figure 3, F and G) and dysfunction of striatal DA metabolism (Supplemental Figure 3A). Of note, as in many other acute PD models (e.g., the 1-methyl-4-phenyl-1,2,3,6-tetrahydropyridine [MPTP] model), α-synuclein–aggregation levels were unaltered (Supplemental Figure 3B), implying that accelerated lipid peroxidation increased the susceptibility of A53T mice to PD. In agreement with the in vitro data, α-synuclein–triggered lipid peroxidation markedly augmented the effect of sorafenib, which only mildly affected GSH content and did not affect MDA or 4-HNE levels in WT mice (Figure 3, H–J). Furthermore, phospholipid profiling showed distinct patterns in these animals (Figure 3L), as noted by accumulation of phospholipid peroxidation products (Figure 3K and Supplemental Figure 3, C and D). Suppression of both RNA and protein levels of GPX4 were also observed in the sorafenib-treated animals (Figure 3, M and N). Collectively, these data suggested that *SNCA* overexpression accelerated ferroptosis through a mechanism that decreased GPX4 activity.

### DAQ modifications induce proteolysis of GPX4 by NEDD4-mediated ubiquitin–protease pathway.

Systemically overexpressing α-synuclein induced a GPX4 decline in the midbrain, but not in the cortex or hippocampus (Figure 1, K and L). Interestingly, midbrain nuclei are distinct from other brain regions in having a DA and iron-rich microenvironment (Supplemental Figure 1M). This may explain the higher susceptibility of the midbrain dopaminergic neurons to oxidative damage, likely through iron-catalyzed autoxidation of DA to DAQ (7). Indeed, we determined that treating PC12 cells with DA and iron significantly increased cytotoxicity (Supplemental Figure 4, A–C), an effect that could be readily rescued by the iron chelator deferiprone (DFP), or GSH (Supplemental Figure 4E). The cytotoxicity of DAQ was deemed to be due to intracellular interactions between DA and iron because BV-2 cells, which lack the DA transporter, showed tolerance (Supplemental Figure 4D). It has been speculated that DAQ covalently binds to the cysteinyl residues in GSH or to proteins, including GPX4 (8). This hypothesis was supported by the observation that, in PC12 cells, DAQ caused a decline in GPX4 protein levels but not *GPX4* mRNA (Supplemental Figure 4G). This decline in GPX4 protein level was reversed in the presence of Fer-1 and DFP (Supplemental Figure 4F). Moreover, the decline in GPX4 levels could be alleviated with GSH (Supplemental Figure 4H), likely suggesting a mechanism that involves DAQ binding to cysteinyl residues.

**Figure 4:**
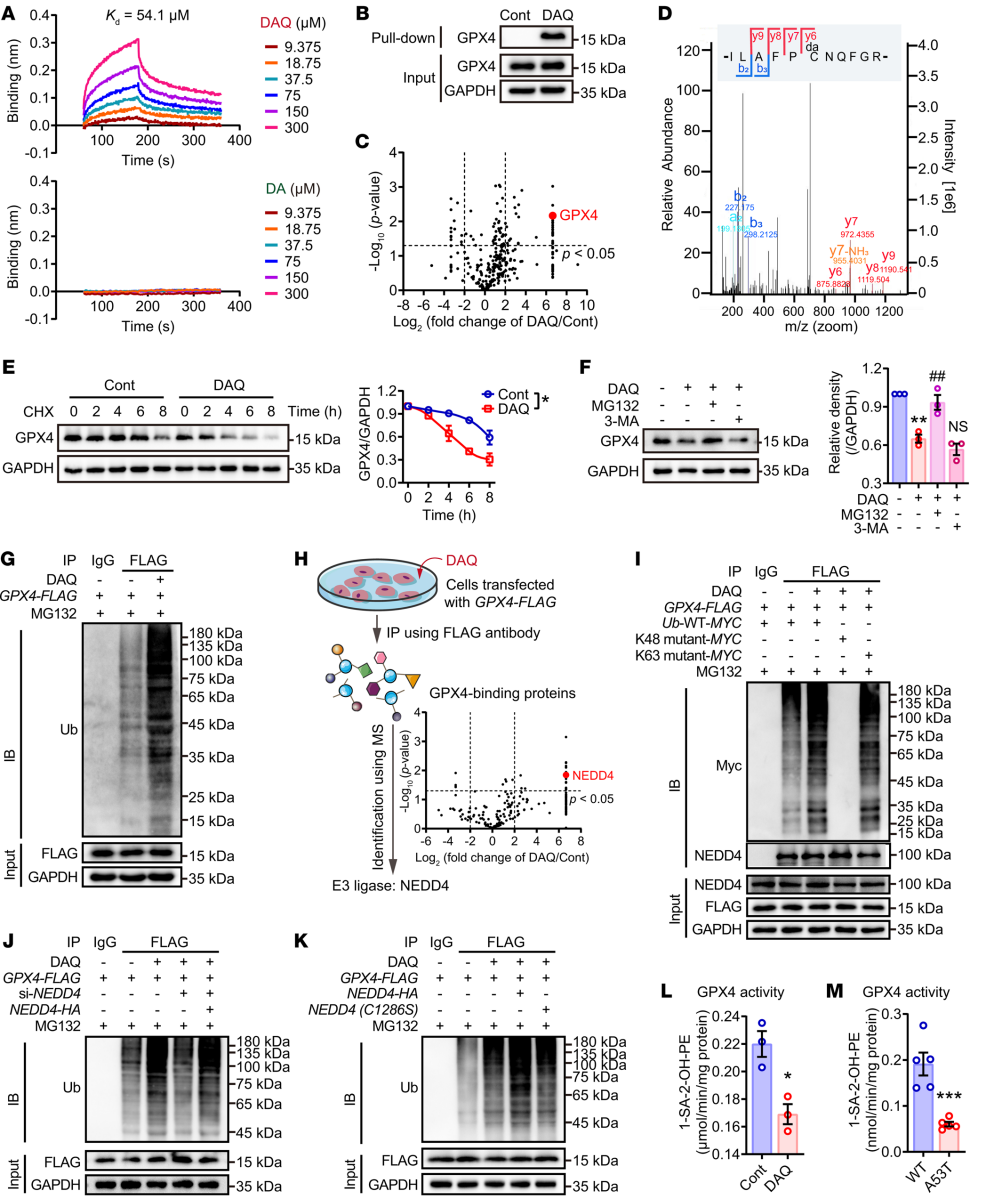
DAQ and α-synuclein contribute to NEDD4-mediated GPX4 ubiquitin proteasome degradation. (**A**) Binding of DAQ to recombinant GPX4 protein was quantitated by BLI. (**B**) The interaction between DAQ (150 μM) and GPX4 in PC12 cells transfected with *GPX4*-expressing plasmid was detected by pull-down assay. (**C**) Proteomic analysis of target proteins pulled down with a DAQ probe and whole cell lysates from PC12 cells. Volcano plots showed the fold changes of DAQ versus control with significance calculated by *t* test. (**D**) LC-MS/MS analysis identified GPX4-Cys102 as the binding site for DAQ. (**E**) GPX4 degradation was assessed by cycloheximide (CHX, 100 μM) chase at indicated time points (left) and the degradation curve was plotted (right) using PC12 cells (*n* = 3 by 2-way repeated measures ANOVA). (**F**) Western blotting showed that MG132 (10 μM) reversed DAQ-induced GPX4 degradation compared with 3-methyladenine (3-MA, 10 mM) in PC12 cells. (**G**) Ubiquitination of GPX4 was determined by coIP using PC12 cells transfected with *FLAG*-tagged *GPX4* plasmid. (**H**) Diagram illustrating the experimental design for identifying GPX4-binding proteins by coIP-MS. (**I**) Ubiquitination of GPX4 and the expression of NEDD4 were examined by coIP using HEK293 cells transfected with plasmids of *FLAG*-tagged *GPX4* and *MYC*-tagged ubiquitin. (**J** and **K**) The role of NEDD4 in GPX4 degradation was verified in HEK293 cells transfected with *NEDD4* siRNA and plasmids of overexpression (*HA*-tagged) or mutation (C1286S). Enzymatic activities of GPX4 in PC12 cells (**L**) or A53T mice (**M**) as analyzed by LC-MS/MS. All data represent mean ± SEM (for cells, *n* = 3, for mice, *n* = 5). *P < 0.05, **P < 0.01 and ***P < 0.001 versus the control group, ^##^*P* < 0.01 versus the DAQ-treated cells, by 1-way ANOVA with Bonferroni’s posthoc test (for **F**), or independent-samples *t* tests (for **C**, **H**, **L**, and **M**).

Binding analysis using bio-layer interferometry (BLI) showed stronger binding of DAQ to GPX4 than to DA (Figure 4A). To verify the direct interactions between DAQ on GPX4, an alkyne-containing DA probe was synthesized and mixed with iron to generate DAQ (Supplemental Figure 4I). PC12 cells were used to optimize intracellular binding time (Supplemental Figure 4J). Pull-down assays performed under these optimal binding conditions confirmed intracellular binding between GPX4 and DAQ (Figure 4B). Proteomic analysis further demonstrated that GPX4 was one of the most prominent proteins covalently modified by DAQ (Figure 4C). Liquid chromatography–mass spectrometry–based (LC-MS/MS-based) analysis localized the site of DAQ binding to GPX4-Cys^102^ (Figure 4D).

An in vitro degradation assay confirmed that DAQ modification accelerated disintegration of GPX4 (Figure 4E), which might account for the observed proteolysis of GPX4 in the cells. This degradation was blocked by the proteasome inhibitor MG132 but not the lysosome inhibitor 3-MA (Figure 4F and Supplemental Figure 4H), suggesting the involvement of the ubiquitin-protease system rather than the autophagy pathway. The presence of polyubiquitination was further confirmed in cells expressing *GPX4* (Figure 4G). Since polyubiquitin linkages via lysine 48 (K48) or 63 (K63) can differentially address proteins for proteasomal degradation or endosome trafficking to the lysosome (19), K48 or K63 point mutation studies were performed to demonstrate that K48 poly ubiquitin chains signaled GPX4 protein for degradation by the proteosome (Figure 4I and Supplemental Figure 4K). In conclusion, the data presented above demonstrated that DAQ modification of GPX4 primed GPX4 for ubiquitination and subsequent degradation.

To further explore the molecular mechanism of DAQ-mediated GPX4 ubiquitination, coimmunoprecipitation (CoIP) MS analysis was performed to screen for potential E3 ubiquitin-protein ligases. The neural precursor cell–expressed developmentally downregulated protein 4 (NEDD4) was identified as the only E3 ubiquitin-protein ligase significantly enriched by DAQ-modified GPX4 (Figure 4H). These data were further confirmed using CoIP Western blotting (Supplemental Figure 4M). In further support of a role for NEDD4 in DAQ-induced GPX4 ubiquitination, the above experiments were performed in cells treated with *NEDD4* siRNA or in cells expressing mutant forms of *NEDD4*. As shown in Supplemental Figure 4L and Figure 4, J and K, ubiquitination was blocked in cells expressing these *NEDD4* variants. The above observations align with findings that NEDD4 was upregulated in α-synuclein–overexpressing A53T mice (Supplemental Figure 4N) and in PC12 cells, which exacerbated GPX4 loss and cell death (Supplemental Figure 4, O and P). Thus, taken together, these results implicated DA and α-synuclein in NEDD4-mediated GPX4 degradation in vitro and in vivo (Figure 4, L and M).

### GPX4 deficiency promotes susceptibility to parkinsonism.

**Figure 5:**
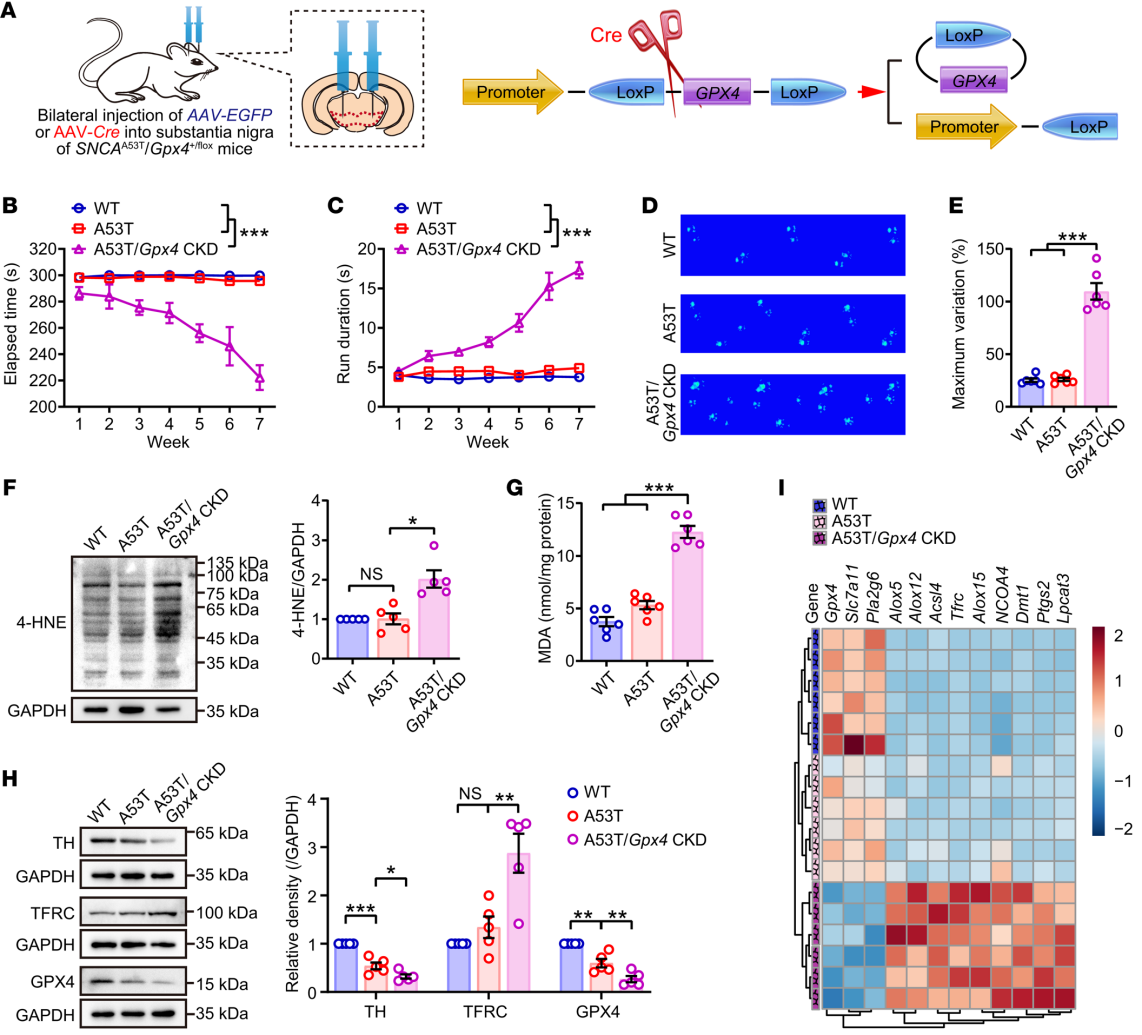
Conditional knockdown of *Gpx4* in substantia nigra accelerates the onset of parkinsonism in *SNCA*^A53T^/*Gpx4*^+/fl^ double transgenic mice. (**A**) Double hemizygous mice were obtained by cross-breeding *SNCA*^A53T^ and *Gpx4*^fl/fl^ *mice*. Schematic diagram showing that 6-month-old *SNCA*^A53T^/*Gpx4*^+/fl^ mice were bilaterally injected with AAV-*Cre* into the substantia nigra to acquire a strain of A53T mice with CKD of *Gpx4*. Behavioral tests (**B**) rotarod and (**C**) pole climbing. Statistics were determined by 2-way repeated measures ANOVA (*n* = 6 mice each group). (**D** and **E**) Catwalk gait analysis (*n* = 6 mice each group). (**F**) Western blotting (left) and quantitative analysis (right) of 4-HNE expression in midbrain (*n* = 5 mice each group). (**G**) Content of MDA in midbrain (*n* = 6 mice each group). (**H**) Western blotting (upper) and quantitative analysis (bottom) of TH, TFRC and GPX4 expressions in midbrain (*n* = 5 mice each group). (**I**) Ferroptosis-related genes were detected by quantitative real-time PCR, and the relative expressions displayed as a heatmap (*n* = 6 mice each group). All data represent mean ± SEM. *P < 0.05, **P < 0.01 and ***P < 0.001, by 1-way ANOVA with Dunnett T3 (for **E**–**G**) or Tukey’s HSD (for **H**) test.

We next investigated the role of GPX4 in PD progression. *SNCA*^A53T^/*Gpx4*^+/fl^ double transgenic mice were bilaterally injected with adeno-associated virus–mediated (AAV-mediated) *Cre* recombinase constructs into the substantia nigra, to conditionally knockdown *Gpx4* expression (A53T/*Gpx4* CKD) before the onset of typical parkinsonism (Figure 5A and Supplemental Figure 5A). Motor behavioral assays revealed that GPX4 deficiency in substantia nigra significantly accelerated parkinsonism progression in A53T mice (Figure 5, B–E, and Supplemental Figure 5, B and C). The animals also showed lipid peroxidation in the midbrain (Figure 5, F and G). Furthermore, reducing vesicular monoamine transporter 2 (VMAT2), exclusively found in neurons and an indicator of PD progression that has been clinically useful for early PD diagnosis (20), suggested that neurons were damaged and DA metabolism was disordered in A53T/*Gpx4* CKD mice (Supplemental Figure 5D), accompanied by disordered DA metabolism (Supplemental Figure 5E). These phenotypes of dopaminergic neuronal dysfunction might be associated with the activation of ferroptosis (Figure 5, H and I) and triggered by the insufficiency of GPX4 during synucleinopathy-associated parkinsonism.

**Figure 6:**
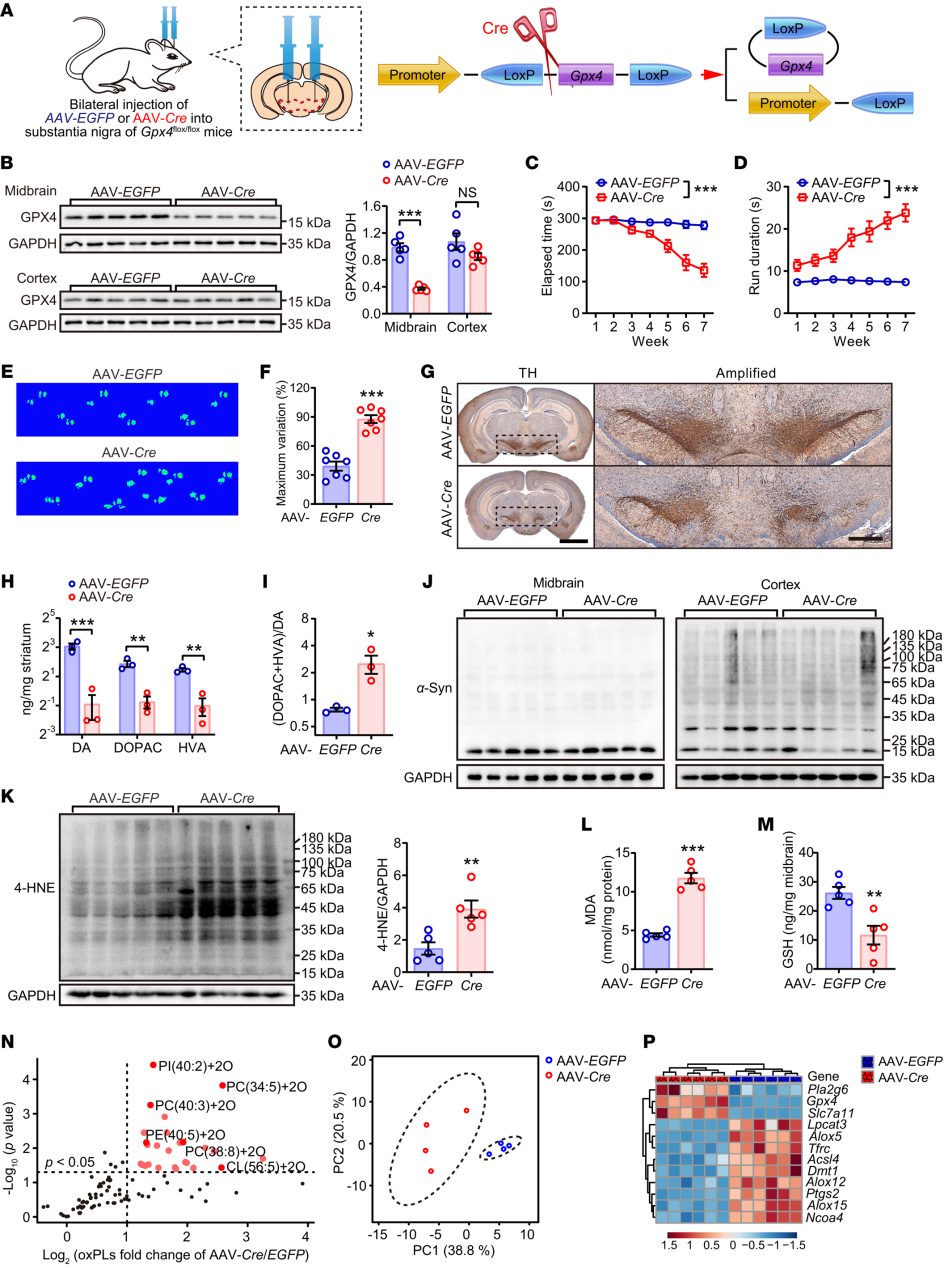
GPX4 is indispensable for maintaining resistance to ferroptosis-associated lipid peroxidation. (**A**) Schematic diagram showing that 8-week-old *Gpx4*^fl/fl^ homozygous mice were bilaterally injected with AAV-*Cre* into the substantia nigra. (**B**) The strain of conditional *Gpx4* knockdown mice acquired from **A** were verified by Western blotting analysis (*n* = 5 mice each group). Disordered motor coordination of *Cre*-injected mice was assessed by behavioral tests, including (**C**) rotarod and (**D**) pole climbing. Statistics were determined by 2-way repeated measures ANOVA (*n* = 7 mice each group). (**E** and **F**) Catwalk gait analysis (*n* = 7 mice each group). (**G**) IHC of coronal brain sections labeled with TH antibody and hematoxylin (left; scale bar: 2 mm). The substantia nigra (dotted area) were amplified on right; scale bar: 500 μm. (**H** and **I**) DA and the metabolites 3,4-dihydroxyphenylacetic acid (DOPAC) and homovanillic acid (HVA) in striatum were quantitated by HPLC-ECD, and the turnover rate of DA and the metabolites were calculated (*n* = 3 mice each group). (**J**) α-Synuclein expression and aggregation was unaffected in both midbrain (left) and cortex (right) in the *Gpx4* CKD mice as determined by Western blotting analysis (*n* = 5 mice each group). (**K**) Western blotting (left) and quantitative analysis (right) of 4-HNE expression in midbrain (*n* = 5 mice each group). The contents of (**L**) MDA and (**M**) GSH were measured in midbrain (*n* = 5 mice each group). The phospholipids in the midbrain were isolated and detected by LC-MS/MS (*n* = 4 mice each group). Data of oxidized phospholipids were extracted and displayed as (**N**) PCA and (**O**) volcano plots showing the fold changes (X-axis) versus significance (Y-axis) by *t* test. (**P**) Ferroptosis-related genes were detected by quantitative real-time PCR assay and the relative expressions were displayed as heatmap (*n* = 6 mice each group). All data represent mean ± SEM. *P < 0.05, **P < 0.01 and ***P < 0.001, by independent-samples *t* tests.

Based on the aforementioned findings showing lipid peroxidation as the causative factor contributing to PD progression in the synuclein-aggregation model, we investigated whether over-activation of lipid peroxidation was sufficient to induce parkinsonism. AAV-*Cre* was bilaterally injected into the substantia nigra of *Gpx4*^fl/fl^ mice (Figure 6A and Supplemental Figure 6A) to obtain conditional *Gpx4* knockout (Figure 6B). These mice exhibited gradually impaired motor coordination functions (Figure 6, C–F, and Supplemental Figure 6, B–H). Dysregulation of TH expression and DA metabolism (Figure 6, G–I) further illustrated that GPX4 deletion led to a severe loss of dopaminergic neurons, which was independent of α-synuclein oligomer aggregation (Figure 6J). Levels of lipid-peroxidation end products increased, and GSH levels decreased in the GPX4 deficient mice (Figure 6, K–M). Additionally, these animals displayed distinct lipid profiles characterized by universal oxidation among almost all phospholipid species (Figure 6, N and O, and Supplemental Figure 6I). In addition, the ferroptosis-related genes were detected in the substantia nigra (Figure 6P), which indicated activation of the ferroptosis signaling pathway.

### GPX4 replenishment alleviates synucleinopathy and parkinsonism.

**Figure 7:**
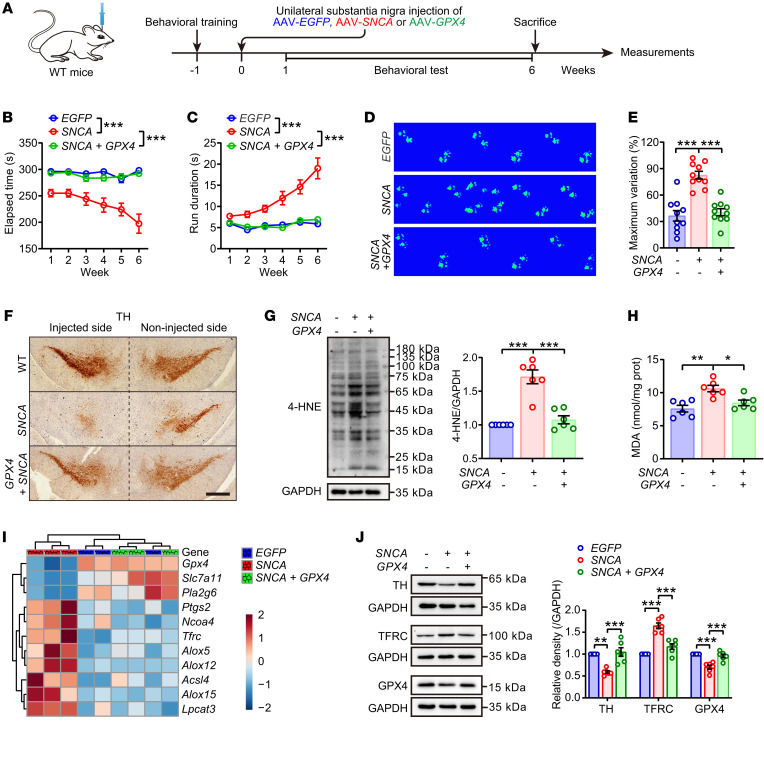
GPX4 attenuated α-synuclein-induced ferroptotic susceptibility. (**A**) Schematic diagram showing that 8-week-old WT mice were unilaterally injected with AAV-loaded human *SNCA* or *GPX4* into the substantia nigra. Disordered motor coordination of *SNCA* mice was mitigated by GPX4, as displayed by behavioral tests (**B**) rotarod and (**C**) pole climbing. Statistics were determined by 2-way repeated measures ANOVA (*n* = 10 mice each group). (**D** and **E**) Catwalk gait analysis (*n* = 10 mice each group). (**F**) IHC of substantia nigra sections labeled with TH antibody and hematoxylin. Scale bar: 500 μm. (**G**) Western blotting (left) and quantitative analysis (right) of 4-HNE expression in midbrain (*n* = 6 mice each group). (**H**) MDA content was measured in midbrain (*n* = 6 mice each group). (**I**) Ferroptosis-related genes were detected by quantitative real-time PCR assay and the relative expressions were displayed as heatmap (*n* = 3 mice each group). (**J**) Western blotting (left) and quantitative analysis (right) of TH, TFRC and GPX4 expressions in midbrain (*n* = 6 mice each group). All data represent mean ± SEM. *P < 0.05, **P < 0.01 and ***P < 0.001, by 1-way ANOVA with Tukey’s HSD (for **E** and **G**) or Bonferroni’s posthoc (for **H**, and **J**) test.

To further confirm the role of GPX4 in ferroptosis and pathogenesis in PD, we tested the effect of GPX4 replenishment in a mouse PD model in which *SNCA* was overexpressed by unilateral injection of AAV-*SNCA* into the substantia nigra. As shown in Figure 7A and Supplemental Figure 7A, GPX4 was overexpressed by local injection of AAV-*GPX4*. GPX4 replenishment not only repressed α-synuclein oligomer formation in the midbrain (Supplemental Figure 7B), but also significantly improved motor coordination functions (Figure 7, B–E). GPX4 effectively ameliorated *SNCA*-related dopaminergic neuron degeneration (Figure 7F) and lipid peroxide accumulation (Figure 7, G and H). Further, GPX4 attenuated activation of ferroptosis and loss of TH expression (Figure 7, I and J)and disrupted dopaminergic neuron and DA metabolism (Supplemental Figure 7, C and D). Taken together, the above observations indicate that GPX4 is the critical factor that protects dopaminergic neurons from ferroptosis and dysfunction, likely via its ubiquitination and subsequent degradation under the direction of DAQ.

## Discussion

PD is a prevalent neurodegenerative disorder that mainly affects people over 65 years old. It has been assumed that the loss of neuronal mass seen in PD is caused by long-term accumulation of iron and lipid peroxides, two features of parkinsonism that are observed in mouse models (21). And, in our study, we also found that loss of neuronal mass took place due to α-synuclein aggregation. In particular, and consistent with recent reports (4, 5), we found that iron accumulation and lipid peroxidation, hallmarks of ferroptosis, were associated with parkinsonism in our mouse models. In support, we established that mild lipid peroxidation induced sorafenib-accelerated PD progress without exacerbating α-synuclein aggregation. Further, substantia nigra–specific *Gpx4* knockdown induced lipid peroxidation and triggered typical parkinsonism, even in the absence of α-synuclein oligomers. These findings suggest that ferroptosis occurs downstream of α-synuclein oligomerization, or synucleinopathy, and contributes to loss of dopaminergic neurons. In addition, the hypothesis of lipid peroxidation–increased PD susceptibility may explain other genetic forms, as well as the most common idiopathic form of PD.

PD has been recognized as a complicated interplay of genetic and environmental factors, both of which affect multiple cellular processes (1). Recent studies have suggested the possible involvement of ferroptosis as a shared downstream mechanism to address such complexities. A primate PD model generated by chronic MPTP injection suggested the participation of ferroptosis in PD pathogenesis (22). A second study indicated that inhibiting ferroptosis was one promising strategy for rescuing MPTP-induced dopaminergic neurodegeneration in a mouse model (23). Lipid peroxidation is a core mechanism involved in the ferroptosis pathway, in which GPX4 acts as the central mediator. Here, we demonstrated that midbrain GPX4 overexpression improved PD-related motor disorders by attenuating oxidative damage and dopaminergic neuron loss. Interestingly, in the early stage of PD, synucleinopathy-related lipid peroxidation and GPX4 downregulation occurred preferentially in the midbrain but not in the cortex or hippocampus. This provides a likely explanation for vulnerability of dopaminergic neurons during PD progression.

To understand why nigral dopaminergic neurons are vulnerable to ferroptosis, we investigated the role of neurotransmitter DA that is locally enriched, although the spatial distribution and precise function of iron is still under investigation (13). It has been speculated that α-synuclein acts in concert with iron and DA to induce the formation of Lewy body in PD (24). Spontaneous oxidation of cytosolic DA generates highly reactive DAQ, which have been shown to bind covalently to cysteinyl residues of proteins such as α-synuclein (25), parkin (26) and DJ-1 (27), thereby disrupting their physiological functions. Furthermore, autoxidation and protein-binding properties of DA are dramatically facilitated by the presence of redox-active transition metal ions such as iron (7). While DA exposure sensitizes cells to metal-induced cytotoxicity through the oxidation of intracellular proteins (28), we determined in this study that DA and iron synergistically exhibited cytotoxicity at their respective subpathological dosage. This cellular toxicity is likely due to direct binding of DAQ to the cysteine residue of GPX4, as supported by LC-MS. DAQ-directed modification is thus largely responsible for dysfunction of GPX4, which is the only known antioxidant enzyme responsible for directly reducing membrane lipid hydroperoxides to maintain cellular integrity and neuronal survival (29). We also determined that, in the presence of DA and iron, glutathione could rescue the deficits caused by GPX4 insufficiency due to its ability to donate cysteine residues to DAQ, essentially preventing GPX4 modification. In summary, DAQ-modification of GPX4 under iron overload and synuclein aggregation form one novel mechanism for dopaminergic neuronal loss in PD.

DA autoxidation can produce neuromelanin (NM), a double membrane granule comprised of melanic, peptide, and lipid components mostly within catecholamine long-lived brain neurons (30, 31). Generally, NM is an effective metal chelator that traps iron and protects neurons from oxidative stress. This equilibrium between iron, dopamine, and NM is crucial for cell homeostasis, and the disturbances of NM synthesis and function could be toxic (32). In humans, NM accumulates with age, which, in turn, is the main risk factor for PD, as NM-containing neurons preferentially degenerate (33). Despite this, it is unclear whether NM plays a role in PD pathogenesis in rodents, because neurons do not form it endogenously. Aside from autooxidation, there are also enzyme-catalyzed metabolic pathways for intracellular DA, including enzymatic deamination by monoamine oxidases (MAOs) into the oxidized DOPAL, which is in turn converted by aldehyde dehydrogenase (ALDH) into DOPAC (34, 35). DOPAL is a cytoplasmic DA intermediate and is the subject of the catecholaldehyde hypothesis for the pathogenesis of PD. Reduced DOPAC/DOPAL ratios indicate decreased ALDH activity (36).

It is believed that GPX4 breakdown is mainly promoted by the autophagy-lysosomal pathway (37), which is probably mediated by chaperone (38) or mechanistic targeting of rapamycin (mTOR) (39). To obtain further insights into GPX4 downregulation, we employed inhibitors of lysosome or proteasome pathways and found, in agreement with a recent study (40), that DAQ-modified GPX4 was mainly degraded through the ubiquitin-proteasome system. We further established that ubiquitination of DAQ-modified GPX4 was mediated by NEDD4, an E3 ubiquitin ligase enzyme involved in the ubiquitination of many membrane proteins, including α-synuclein (41). In close agreement with our findings of α-synuclein-induced NEDD4 upregulation, a previous study revealed that the elevation of NEDD4 was more abundant in cells overexpressing A53T (42). Given that GPX4 deficiency is induced by DAQ-modified ubiquitination via α-synuclein–induced elevation of NEDD4 levels, a model is proposed in which α-synuclein, DA, and iron collaborate to aggravate ferroptosis in midbrain nuclei, causing dopaminergic neuronal loss during PD development. Given these findings, preservation of GPX4 activity may form a promising strategy for early intervention of PD.

In summary, the work presented here demonstrates a novel pathway in which iron-induced DA oxidation disrupted GPX4 function by sequestering it for degradation via the ubiquitin-proteosome system, leading to unfettered lipid peroxidation that culminated in ferroptosis. Due to the relative abundance of DA in midbrain nuclei, our findings provide what we believe to be new insights for explaining the vulnerability of nigral dopaminergic neurons in PD progression. Therefore, strategies that disrupt autooxidation, including approaches that improve the antioxidant status of dopaminergic neurons, hold promise in alleviating the effects of this disease.

## Methods

### Animal care

WT, *SNCA*^A53T^ (Jackson Laboratory, stock no. 004650, referred to as A53T), and *Gpx4*^fl/fl^ (Jackson Laboratory, stock no. 027964) C57BL/6J mice were housed in groups in cages at a mean constant temperature (23 ± 2^o^C), humidity (55% ± 5%) and illumination (12 hour light–dark cycle), with free access to standard pellet chow and water. Mice were adapted to the facilities for 1 week before experiments began.

### Interventions in transgenic mice by virus injection

#### Virus injection.

Mice were anesthetized by isoflurane (3% for induction and 1.5% for maintenance). After local shaving, sterilization, and incision of head skins, the substantia nigra (3.8 mm posterior to bregma and 1.5 mm lateral from the midline) was localized under a stereotaxic instrument (RWD Life Science Inc). After drilling a hole by high-speed microdrill, 200 nL of AAV serotype 2/9 (viral titer, > 1×10^13^ genome copies/mL, Brain VTA) was injected using a glass micropipette connected to an ultra-micro injection pump (Nanoliter 2010, World Precision Instruments) with a flow rate of 70 nL/min. The micropipette was retained for 10 minutes before retraction. The head skin was closed for postsurgery monitoring. After the surgery, all mice were housed to recover for at least 7 days with free access to food and water before behavioral training.

#### A53T/Gpx4 CKD mice.

*SNCA*^A53T^ and *Gpx4*^fl/fl^ were crossbred to obtain *SNCA*^A53T^/*Gpx4*^+/fl^ double transgenic hemizygous mice. Mice were given bilateral injections into the substantia nigra with AAV expressing *Cre*-*EGFP* under the control of a human synapsin-1 promoter (AAV-*hSyn*-*Cre*-*EGFP*), as described above, to obtain a strain of A53T with conditional knockdown of *Gpx4* (CKD). The control mice were injected AAV-*hSyn-EGFP*.

#### Gpx4 CKD mice.

*Gpx4*^fl/fl^ homozygous were bilaterally injected AAV-*hSyn*-*Cre*-EGFP into the substantia nigra as described above to obtain a strain of *Gpx4* CKD mice. The control mice were injected AAV-*hSyn-EGFP*.

#### SNCA + GPX4 OE mice.

WT mice were unilaterally injected with AAV-*hSyn*-*SNCA* or -*GPX4* into the substantia nigra as described above to obtain strains overexpress human α-synuclein, GPX4, or both. The control mice were injected with AAV-*hSyn-EGFP*.

### Cell lines

PC12 cells carrying doxycycline-inducible *SNCA*^A53T^ were maintained in DMEM (high glucose) medium containing 10% heat-inactivated horse serum and 5% FBS. HEK293 cells were maintained in DMEM (high glucose) medium containing 10% FBS. Both cell lines were cultured in incubators with controlled temperatures of 37°C, 5% CO_2_, and 95% humidity.

### Behavioral tests

The motor coordination of the mice was assessed by rotarod test, pole test, and gait analysis, as described previously (5, 43). Additional information is available in Supplemental Methods.

### TH, GPX4, or 4-HNE staining of brain section

IHC and immunofluorescence was conducted as described previously (43). Additional information is available in Supplemental Methods.

### LC-MS analysis for determination of GSH, DA, DOPAC, and HVA

The substantia nigra and striatum were obtained on ice, weighed, recorded, and stored at –80°C. The substantia nigra were prepared for GSH detection, and the striatum were prepared for DA, DOPAC, and HVA detection, as described previously (5). Additional information is available in Supplemental Methods.

### MDA measurement

MDA was determined by the TBARS assay according to the protocol provided by the manufacturer (Beyotime Biotechnology). Briefly, the midbrain tissues were obtained and homogenized on ice, and the supernatants were collected and detected using an ELISA plate reader (Thermo Fisher Scientific). The concentration of MDA was calculated according to the standard curve, which was prepared in advance.

### Iron measurement

The relative iron concentration in midbrain tissues of mice was determined using an Iron Assay Kit according to the protocol provided by the manufacturer (Abcam).

### Western blotting analysis

Tissues or cells were resuspended in lysis buffer on ice for 5 minutes, and the supernatants were collected after centrifugation at 13,000*g* for 15 minutes. The protein was determined using the Pierce BCA protein assay kit (Thermo Fisher Scientific). Protein lysates (30 μg) were separated in 10% or 15% SDS-PAGE and transferred onto Immobilon-P PVDF membrane (Sigma-Aldrich). Bands were detected using Pierce ECL Western blotting Substrate (Thermo Fisher Scientific).

### Quantitative real-time PCR

Total RNA was extracted from mice midbrain tissues using TRIzol reagent according to the protocol provided by the manufacturer (TaRaKa). RNA concentrations were determined by OD measurement at 260 nm on a spectrophotometer (Implen), and cDNA was synthesized from the purified RNA by both random and oligo (dT) priming using an iScript cDNA synthesis kit (TIANGEN). RNA levels were measured using the SYBR green method (TOYOBO) on a reverse transcription machine (CFX Connect, Bio-Rad) and the relative quantitation method.

### Cell viability assessment

Cells were dispensed in dishes at a density of 5 × 10^5^ cells. After treatment, cells were incubated with SYTOX Green according to the manufacturer’s instructions (Thermo Fisher Scientific). The cells were then collected for analysis in a BD FACSCanto II flow cytometer (BD Biosciences). The excitation and emission wavelengths of SYTOX Green are 488 nm and 523 nm, respectively.

### Lipid peroxidation assessment

Cells were dispensed in confocal microscopy compatible dishes at a density of 5 × 10^5^ cells. After treatment, cells were incubated with C11-Bodipy for 30 minutes at 37°C. Then the cells were collected for analysis in a BD FACSCanto II flow cyt or imaged using a ZEISS LSM 800 confocal laser scanning microscope (Carl Zeiss). The excitation and emission wavelengths of C11 are 581 nm and 591 nm, respectively. The excitation and emission wavelengths of oxidized C11 are 500 nm and 510 nm, respectively.

### LC-MS/MS-based phospholipidomics analysis of plasma membrane lipids

Preparation of membrane lipids and phospholipidomics analysis was performed as described previously (44). Additional information is available in Supplemental Methods.

### GPX4 recombinant expression and purification

A full-length rat *Gpx4* gene was cloned into a modified pRSF-Duet vector preceded by a His6-SUMO tag. Protein expression was performed in *E*. *coli* strain BL21 (DE3). After initial growth to an OD600 of 0.8, cells were induced using 0.4 mM isopropyl β-D-1-thiogalactopyranoside (IPTG) and cultured at 20°C for 16 hours. Cells were harvested by centrifugation and lysed in buffer containing 50 mM Tris-HCl (pH 8.0), 1 M NaCl, 25 mM imidazole, 0.5 mM β-mercaptoethanol, 1 mM PMSF, and protease inhibitors. The fusion proteins were purified using a Ni-NTA column. The proteins were washed with a wash buffer containing 25 mM imidazole, 1 M NaCl, and 25 mM Tris at pH 8.0. GPX4 protein was eluted with an elution buffer containing 250 mM imidazole, 1 M NaCl, and 25 mM Tris at pH 8.0. The His6-SUMO tag was cleaved by ULP1 treatment, and the tag-free protein was fractioned with Ni-NTA column and Superdex 75–size exclusion column (GE Healthcare) with 25 mM HEPES (pH 8.0), 300 mM NaCl, and 1% glycerol. The final purified GPX4 protein was stored at –80°C.

### BLI

BLI kinetic experiments were performed using an OctetRED96 instrument from PALL/ForteBio. The assay was completed in PBS (pH 7.4) as assay buffer with 0.1% BSA and 0.01% Tween-20 added to reduce nonspecific interactions and 2% DMSO added to increase compound solubility. Biotinylated GPX4 protein prepared by Thermo EZ-Link long-chain biotinylation reagent was connected to a Super Streptavidin (SSA) biosensor by immersing the sensor in a 96-well black plate loaded with protein solution, the concentration of which was predetermined by preliminary control experiments to achieve the best signal-to-noise ratio. Protein-conjugated sensors were moved and immersed into wells containing pure assay buffer and equilibrated in the buffer for 10 minutes to eliminate loose, nonspecific binding proteins and establish a stable baseline. Association and dissociation times were carefully determined to ensure complete association and dissociation. A DMSO-only reference was included in all assays. Raw kinetic data collected were processed by the manufacturer’s supplied data analysis software using a dual reference subtraction method where both the DMSO-only reference and the inactive protein reference were subtracted. The resulting data were analyzed based on a 1:1 binding model, from which *K*_on_ and *K*_off_ values were obtained, and then K*_d_* values were calculated.

### Synthesis of DA probes

Pentynoic acid (1.0 mmol) dissolved in dichloromethane (10 mL) before EDCI (1.0 mmol) was added, and the reaction was carried out at 25°C for 30 minutes. Then 3,4-dimethoxyphenethylamine was added (1.25 mmol) and the solution cooled to 0°C before dichloromethane solution containing 4-DMAP (40 mg) was added dropwise to the reaction system, which was maintained at 0°C for 1 hour. The reaction was continued at 25°C overnight. The reaction mixture was quenched with water (10 mL) and extracted with dichloromethane (10 mL, × 2). The organic phases were combined, washed twice with saturated brine, dried over anhydrous sodium sulfate, and purified to obtain a white solid product S1 (240.4 mg, purity 92%).

S1 (240.4 mg, 0.92 mmol) was dissolved in anhydrous dichloromethane (10 mL), cooled to 0°C, and BBr3 was added (553.5 mg, 2.3 mmol), and reacted for 4 hours. The reaction mixture was quenched with water (10 mL) and extracted with dichloromethane (10 mL, × 2). The organic phases were combined, washed twice with saturated brine, dried over anhydrous sodium sulfate, and purified to obtain alkyne-containing DA probe (DA-yne, 171.7 mg, purity 80%).

The following is information regarding the 1H-NMR spectrum and 13C-NMR spectrum of the probe: 1H NMR (300 MHz, MeOD) δ 6.56 (dd, J = 10.8, 5.0 Hz, 2H), 6.41 (dd, J = 8.0, 2.1 Hz, 1H), 3.32-3.14 (m, 2H), 2.52 (t, J = 7.4 Hz, 2H), 2.41-2.29 (m, 2H), 2.28-2.20 (m, 2H), 2.14 (t, J = 2.6 Hz, 1H). 13C NMR (300 MHz, MeOD) δ 172.6, 144.8, 143.4, 130.7, 119.7, 115.5, 115.0, 82.2, 81.7, 41.0, 34.7, 34.6 14.4. HRMS (ESI) calcd. for C_13_H_15_NO_3_ 234.1125 [M + H]^+^, found 235.1194.

### Pull down assay for GPX4

PC12 cells were treated with DAQ probe (DA-yne probe contains FeCl_3_ solution) or DMSO for 6 hours, and then were harvested and lysed with RIPA buffer supplemented with protease inhibitor cocktails and phosphatase inhibitor cocktails. A freshly premixed click chemistry reaction cocktail (50 μM TAMRA-N_3_ from 25 mM stock solution in DMSO, 100 μM TBTA from 50 mM stock solution in DMSO, 1 mM TCEP from 1 M stock solution in deionized water, and 1 mM CuSO_4_ from 1 M stock solution in deionized water. All the stock solutions above were freshly prepared.) was added to equal amounts of protein lysate. The reaction mixture was further incubated at 25°C for 2 hours, and the solution was transferred to a centrifugation tube where prechilled acetone at –20°C was added and the solution was then dissolved in PBS **+** 1% SDS. Upon incubation with streptavidin beads at 25°C for 4 hours, 2 × 100 μL SDS loading buffer was added and heated at 95°C for 10 minutes. Proteins were separated by SDS-PAGE gel and transferred to Immobilon-P PVDF membrane (Sigma-Aldrich). Protein expression was detected using GPX4 antibody and visualized using secondary antibody conjugated with HRP and Pierce ECL Western blotting substrate (Thermo Fisher Scientific) as the substrate of HRP. The immunoblot was detected by Tanon 5200 Chemiluminescence Image Analysis System (Tanon Science & Technology).

### Modification site analysis of DAQ to GPX4 protein

GPX4 protein was diluted to 1 mg/mL with PBS and incubated with DAQ (DA + FeCl_3_, 150 μM) at 37°C for 2 hours. Protein was subsequently collected by centrifugation (14,000*g* at 4°C for 20 minutes) and the supernatant was transferred to an ultrafiltration membrane spin column and centrifuged at 12,500*g* for 5 minutes. The bound protein was washed with tetraethylammonium bromide (TEAB) buffer, followed by incubation with DTT (1 M) at 37°C for 45 minutes and 3-indoleacetic acid (IAA, 1 M) at 25°C for 1 hour. The bound protein was washed with TEAB buffer and digested with trypsin overnight. Peptide-containing supernatant was collected by centrifugation (12,500*g* for 5 minutes) and desalted on a C18 column. The samples were analyzed by LC-MS/MS.

### Cycloheximide chase assay

The protein stability of GPX4 was measured by cycloheximide pulse chase assay. After treatment, the PC12 cells were added with 100 μM cycloheximide to interfere with the translocation step in protein synthesis. Cells were collected at indicated time points for Western blotting analysis.

### Proteomics for E3 ligase

PC12 cells were pretreated with the proteasome inhibitor MG132 (10 μM) for 4 hours after transfection with GPX4-FLAG plasmid for 24 hours. DAQ (DA + FeCl_3_, 150 μM) was added 6 hours before harvest. The cell lysates were centrifuged at 12,000*g* for 10 minutes and the supernatant was mixed with the FLAG antibody overnight at 4°C on a shaker, before incubation with prewashed protein A/G agarose beads at 25°C for 4 hours. The beads were collected and washed, and the bound proteins were eluted by Laemmli buffer containing 500 μL 6 M urea, 25 μL 200 mM DTT, and 25 μL 500 mM IAA in the dark at 25°C for 30 minutes. The eluent was incubated with 150 μL 2 M urea, 150 μL 1 mM CaCl_2_, and 1 μL trypsin (1 μg/μL) at 37°C overnight. After that, the protein samples were purified by ODS C18 SPE column (Agilent) and analyzed by LC-MS/MS, equipped with an EASY-nLC 1200 HPLC system and Orbitrap Fusion Lumos mass spectrometer (Thermo Fisher Scientific). For LC separation, tryptic peptides were sequentially injected into an Acclaim PepMap 100 C18 column (100 μM × 2 cm, 5 μM, Thermo Fisher Scientific) and an Acclaim PepMap 100 C18 column (50 μM × 15 cm, 2 μM, Thermo Fisher Scientific).

### CoIP assay

Cells were harvested and lysed with RIPA buffer supplemented with protease inhibitor cocktails and phosphatase inhibitor cocktails. Cell lysates were prepared for immunoprecipitations and 5% lysis buffer from each sample was used as the input control. The supernatant was transferred to a fresh tube and incubated with the primary antibodies overnight on a rotator at 4°C. The samples were then incubated with protein A/G agarose beads at 25°C for 2 hours. The beads were collected and washed, and the bound proteins were eluted by heating at 95°C for 10 minutes in 50 μL SDS loading buffer. The immunoprecipitants were separated by SDS-PAGE and analyzed by Western blotting.

### GPX4 activity assessment

PC12 cells expressing GPX4-FLAG were pretreated with DA and FeCl_3_ solution for 6 hours. Midbrain tissues were soaked in cold PBS and sonicated for 30 seconds on ice. All samples were lysed with 3 flash-freeze-thawing cycles using –80°C and 37°C, respectively. The supernatants were collected for further protein quantification using BCA protein assay kit after centrifugation at 12,000*g* at 4°C for 15 minutes. The supernatants (100 μg protein) were mixed with 100 mM Tris-HCl containing 5 mM EDTA, 0.1% Triton X-100, 1.5 mM NADPH, 3 mM GSH, and 10 μM 1-SA-2-15-HpETE-PE, in the absence or presence of 10 μM RSL3, and incubated at 37°C for 30 minutes. The reaction was stopped by the addition of chloroform and methanol (2:1, v/v) followed by vortexing for 2 minutes. The chloroform-methanol layer was collected, dried under nitrogen, and redissolved in methanol for LC-MS analysis. GPX4 activity was quantified as nmols of catalytic product 1-SA-2-OH-PE/min/mg of protein.

### Statistics

All data were expressed as mean ± SEM of independent experiments. The data were analyzed by IBM SPSS Statistics 25.0 (SPSS Inc). *P* values were determined by 2 tailed independent-samples *t* test, 1-way ANOVA with Bonferroni, Dunnett T3, Tukey HSD, and LSD posthoc tests, and 2-way repeated measures. *P* < 0.05 is considered statistically significant.

### Study approval

All animal experiments were performed in accordance with the National Institutes of Health’s Guide for the Care and Use of Laboratory Animals (NIH publication no. 80-23, revised in 1996) and were approved by the Animal Ethics Committee of Jinan University (Approval no. 20130904001). The methods used for the supplemental figures are described in the Supplemental Methods. Information on reagents, antibodies, plasmids, and other materials used in this study can be found in the Supplemental Lists of Resources.

## Author contributions

JS conducted most of the in vitro experiments and acquired and analyzed the core data, incuding BLI kinetic experiments, synthesis of DA probes, modification site analysis of DAQ to GPX protein, proteomics for E3 ligase, and most of the LC-MS/MS-based phospholipidomics, and thus listed as the first co–author. XML conducted most of the in vivo experiments and acquired and analyzed data, including interventions in transgenic mice by virus injection, behavioral tests, and part of phospholipidomics, and thus listed as the second co-author. DHL conducted most of the in vivo experiments and acquired and analyzed data, including behavioral tests and part of phospholipidomics, and thus listed as the third co–author. DHL conducted most of the in vivo experiments and acquired and analyzed data. MW assisted in conducting in vivo experiments and acquiring data. KL assisted in conducting in vitro experiments and acquiring data. SRL assisted in conducting modification site analysis of DAQ to GPX4 protein. ZQL supervised and assisted in modification site analysis of DAQ to GPX4 protein. CJZ conducted the preparation of GPX4 recombinant. ZMZ supervised and conducted the preparation of GPX4 recombinant. CYY assisted in conducting in vivo experiments and acquiring data. MHP assisted in conducting in vivo experiments and acquiring data. HBG conducted the LC-MS/MS-based experiments and acquired data. JCF conducted the LC-MS/MS-based experiments and acquired data. YFC supported the LC-MS platform and consulted regarding the data analysis. FH supervised and advised the project. WYS conducted the LC-MS/MS-based experiments and analyzed data. HK supervised and advised the project. YFL advised the project. WJD analyzed and approved data, prepared all the figures and tables, and wrote the manuscript. GLJ supervised and advised the project. LZ advised the project and revised the manuscript. RRH designed and supervised the project, revised and approved the manuscript.

## Supplementary Material

Supplemental data

## Version 1 05/15/2023

Electronic publication
