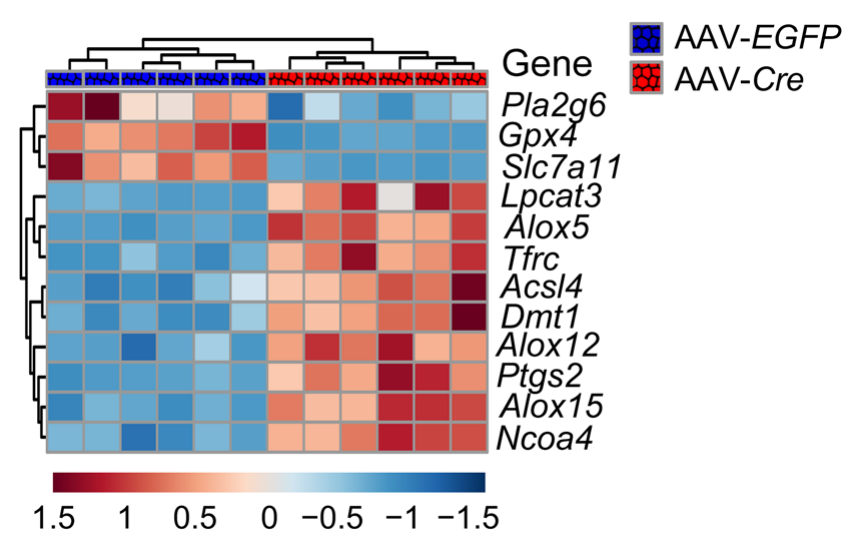# Midbrain dopamine oxidation links ubiquitination of glutathione peroxidase 4 to ferroptosis of dopaminergic neurons

**DOI:** 10.1172/JCI173110

**Published:** 2023-07-03

**Authors:** Jie Sun, Xiao-Min Lin, Dan-Hua Lu, Meng Wang, Kun Li, Sheng-Rong Li, Zheng-Qiu Li, Cheng-Jun Zhu, Zhi-Min Zhang, Chang-Yu Yan, Ming-Hai Pan, Hai-Biao Gong, Jing-Cheng Feng, Yun-Feng Cao, Feng Huang, Wan-Yang Sun, Hiroshi Kurihara, Yi-Fang Li, Wen-Jun Duan, Gen-Long Jiao, Li Zhang, Rong-Rong He

Original citation: *J Clin Invest*. 2023;133(10):e165228. https://doi.org/10.1172/JCI165228

Citation for this corrigendum: *J Clin Invest*. 2023;133(13):e173110. https://doi.org/10.1172/JCI173110

In the original version of Figure 6P, the labels for AAV-*EGFP* and AAV-*Cre* were inverted. The correct figure part is below. The HTML and PDF files have been updated online. The authors have stated that this correction does not change any of the conclusions made.

The authors regret the error. 

## Figures and Tables

**Figure:**