# Supplementary material for: Midbrain dopamine oxidation links ubiquitination of glutathione peroxidase 4 to ferroptosis of dopaminergic neurons
Source: J Clin Invest. 2023 May 15;133(10):e165228. doi: 10.1172/JCI165228 (PMC10178840; doi:10.1172/JCI165228)
Supplement: Supplemental data [file jci-133-165228-s182.pdf]

Supplementary material

Midbrain dopamine oxidation links ubiquitination of glutathione peroxidase 4 to ferroptosis of dopaminergic neurons

Jie Sun [a,1], Xiao-Min Lin [a,1], Dan-Hua Lu [a,1], Meng Wang [1], Kun Li [1], Sheng-Rong Li [1], Zheng-Qiu Li [1], Cheng-Jun Zhu [1], Zhi-Min Zhang [1], Chang-Yu Yan [1], Ming-Hai Pan [1], Hai-Biao Gong [1], Jing-Cheng Feng [1], Yun-Feng Cao [3], Feng Huang [5], Wan-Yang Sun [1], Hiroshi Kurihara [1], Yi-Fang Li [1], Wen-Jun Duan[*,1], Gen-Long Jiao[*,1], Li Zhang[*,4], Rong-Rong He[*,1,2,5]

1. The First Affiliated Hospital of Jinan University/Guangdong Engineering Research Center of Chinese Medicine & Disease Susceptibility/International Cooperative Laboratory of Traditional Chinese Medicine Modernization and Innovative Drug Development of Chinese Ministry of Education (MOE)/Guangdong Province Key Laboratory of Pharmacodynamic Constituents of TCM and New Drugs Research/The Sixth Affiliated Hospital of Jinan University, Jinan University, Guangzhou 510632, China
2. State Key Laboratory of Quality Research in Chinese Medicine, Macau University of Science and Technology, Macau, China.
3. Shanghai Institute for Biomedical and Pharmaceutical Technologies, NHC Key Laboratory of Reproduction Regulation, Shanghai 200032, China
4. Key Laboratory of CNS Regeneration (Ministry of Education), Guangdong-Hong Kong-Macau Institute of CNS Regeneration, Jinan University, Guangzhou 510632, China
5. School of Chinese Materia Medica and Yunnan Key Laboratory of Southern Medicinal Utilization, Yunnan University of Chinese Medicine, Kunming 650500, China

[a]Contributed equally to this work.

[*]Corresponding to:

Email: rongronghe@jnu.edu.cn, Tel & Fax: +86-20-85221559, zhangli@jnu.edu.cn, jiaogenlong@163.com, duanwj@jnu.edu.cn.

The authors declare no conflict of interest.

Content

1. Supplementary figures and figure legends, Page 1-16;
2. Supplementary methods, Page 17-22;
3. Lists of resources, Page 23-26;
4. Reference, Page 27.

Supplementary figures and figure legends

Supplementary figure 1

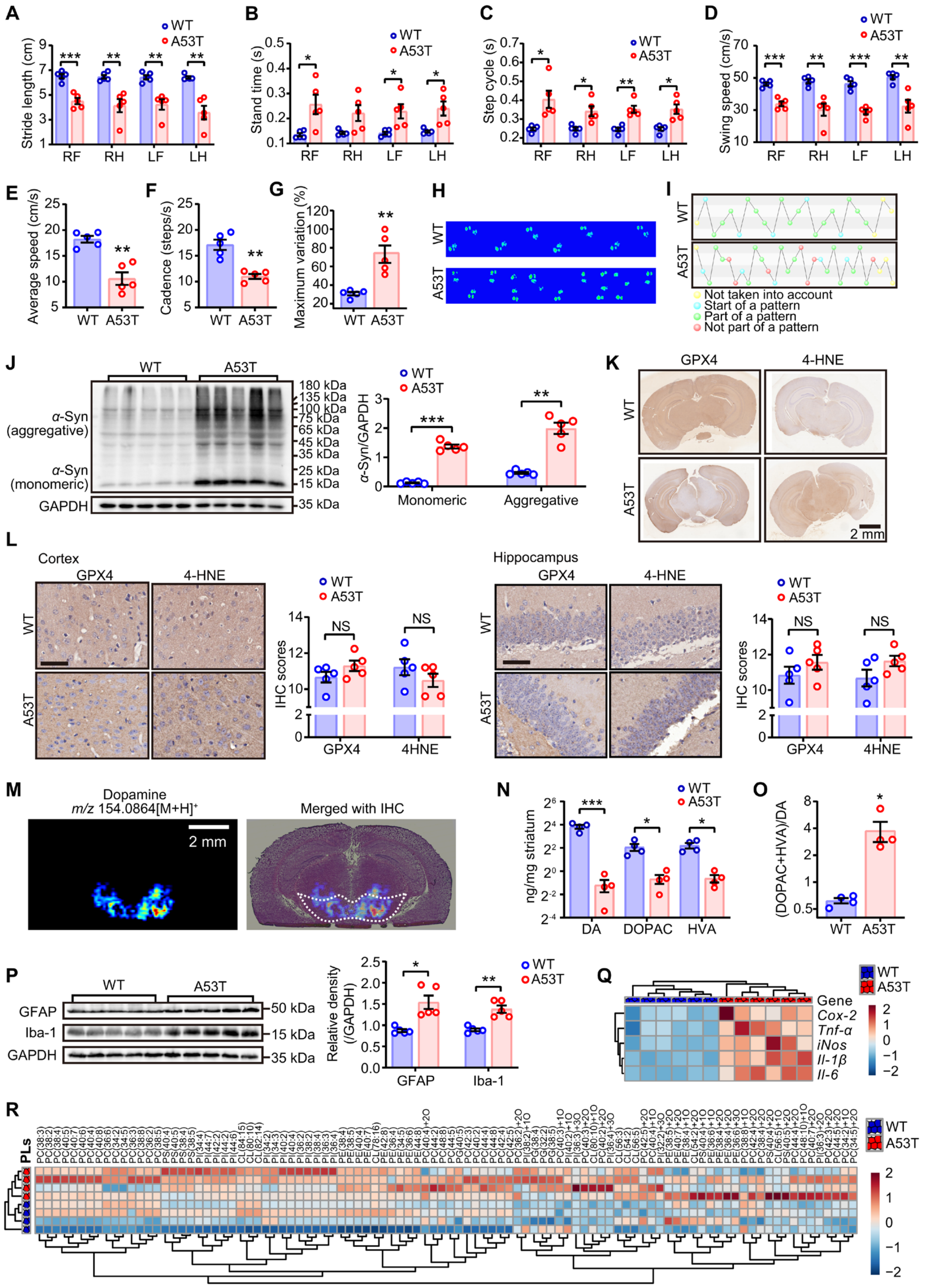

Figure S1. PD progression is combined with phospholipid oxidation in *SNCA*^{A53T} transgene mice. Disordered motor coordination of A53T mice compared to WT mice was assessed by Catwalk gait analysis including the (**A**) stride length, (**B**) stand time, (**C**) step cycle, (**D**) swing speed, (**E**) average

speed, (**F**) cadence and (**G**) maximum variation (*n* = 5 mice each group). The gait pattern, referring to the order in which the mice complete a sequence of walking, was visualized as (**H**) footprint view and (**I**) footfall pattern. RF, right front. RH, right hind. LF, left front. LH, left hind. Green balls: normal pattern. Red balls: abnormal pattern. Blue balls: the beginning of a walking cycle. Yellow balls: data that were excluded from the test. (**J**) *α*-Synuclein expression and aggregation was determined by Western blotting analysis (*n* = 5 mice each group). (**K**) IHC of coronal brain sections labeled with GPX4 and 4-HNE antibodies and hematoxylin. (**L**) IHC GPX4 and 4-HNE in the cortex and hippocampus in A53T mice. IHC magnification (left) and IHC score (right). Scale bar: 50 μm. (**M**) The dopamine (DA) in substantia nigra (dotted area) was detected by AFADESI-MSI assay in positive ion mode. Representative image was merged with H&E-stained coronal section. (**N-O**) The contents of DA and the metabolites 3,4-dihydroxyphenylacetic acid (DOPAC) and homovanillic acid (HVA) in striatum were determined by LC-MS, and the turnover rate of DA and the metabolites were calculated (*n* = 4 mice each group). (**P**) The inflammation of midbrain was determined by Western blotting analysis displaying the expressions of GFAP and Iba-1 (*n* = 5 mice each group). (**Q**) Inflammation-related genes were detected by quantitative real-time PCR assay and relative expressions were displayed as heatmap (*n* = 6 mice each group). *Cox-2,* cyclooxygenase-2. *Tnf-α,* tumor necrosis factor-*α*. *iNos,* inducible nitric oxide synthase. *Il-1β,* interleukin-1β. *Il-6,* interleukin-6. (**R**) Data of phospholipids were extracted and displayed as heatmap (*n* = 4 mice each group). PL, phospholipid. PC, phosphatidylcholine. PE, phosphatidylethanolamine. PI, phosphatidylinositol. PS, phosphatidylserine. CL, cardiolipin. All data represent mean ± SEM. $^{*}p < 0.05$, $^{**}p < 0.01$ and $^{***}p < 0.001$, by independent-samples *t*-test.

Supplementary figure 2

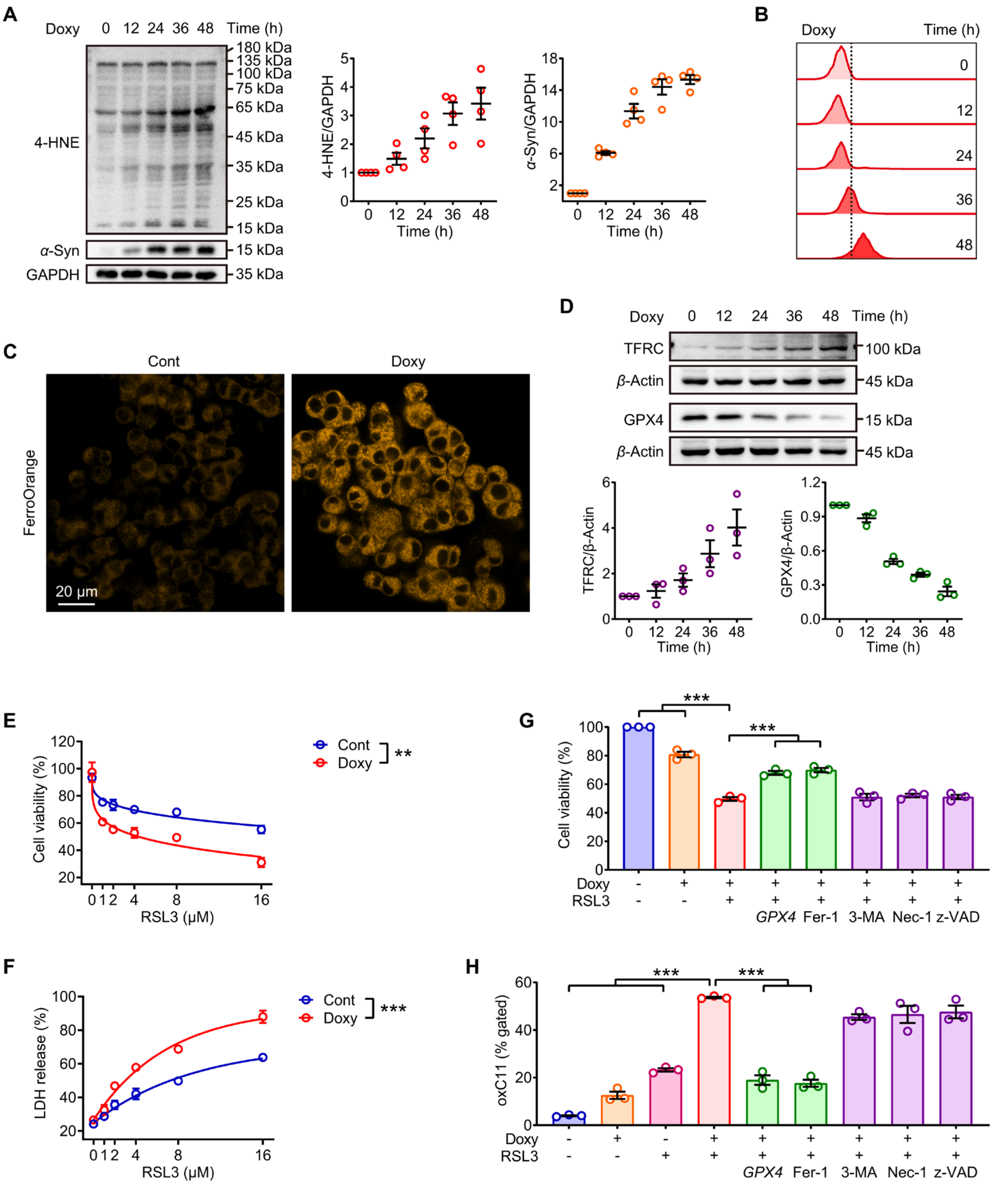

Figure S2. *α*-Synuclein overexpression leads to lipid peroxidation and ferroptotic susceptibility in PC12 cell line. PC12 cell line carrying doxycycline-inducible *SNCA*^{A53T} were utilized for *α*-synuclein overexpression *in vitro*. (**A**) Western blotting (left) and quantitative analysis (right) of 4-HNE and *α*-synuclein expressions in PC12 cells treated with doxycycline for 12, 24, 36

and 48 hours (n = 4 independent samples). (**B**) *SNCA*-overexpression-induced lipid peroxides were labelled with Liperfluo and measured by flow cytometry. (**C**) Cytoplasmic labile iron was stained by FerroOrange. (**D**) Western blotting (upper) and quantitative analysis (bottom) of TFRC and GPX4 expressions in PC12 cells treated with doxycycline for 12, 24, 36 and 48 hours. *SNCA*-overexpressed PC12 cells were treated with RSL3 (12.5 μM) for 1, 2, 4, 8 and 16 hours, and (**E**) cell viability was determined by MTT assay and (**F**) plasma membrane integrity was determined by LDH assay. $^{**}p < 0.01$ and $^{***}p < 0.001$, by two-way repeated measures ANOVA. *SNCA*-overexpressed PC12 cells were treated with indicated agents for 12 h. *GPX4*, plasmid expressing GPX4. Fer-1, ferrostatin-1, 10 μM. 3-MA, 3-methyladenine, 10 mM. Nec-1, necrostatin-1, 5 μM. z-VAD, z-VAD-FMK, 20 μM. RSL3, 12.5 μM. The (**G**) Cell viability was determined by MTT assay, and (**H**) lipid peroxides were labelled with ox-C11 and determined by flow cytometry. All data represent mean ± SEM (n = 3 independent samples). $^{**}p < 0.01$ and $^{***}p < 0.001$, by one-way ANOVA with Bonferroni test.

Supplementary figure 3

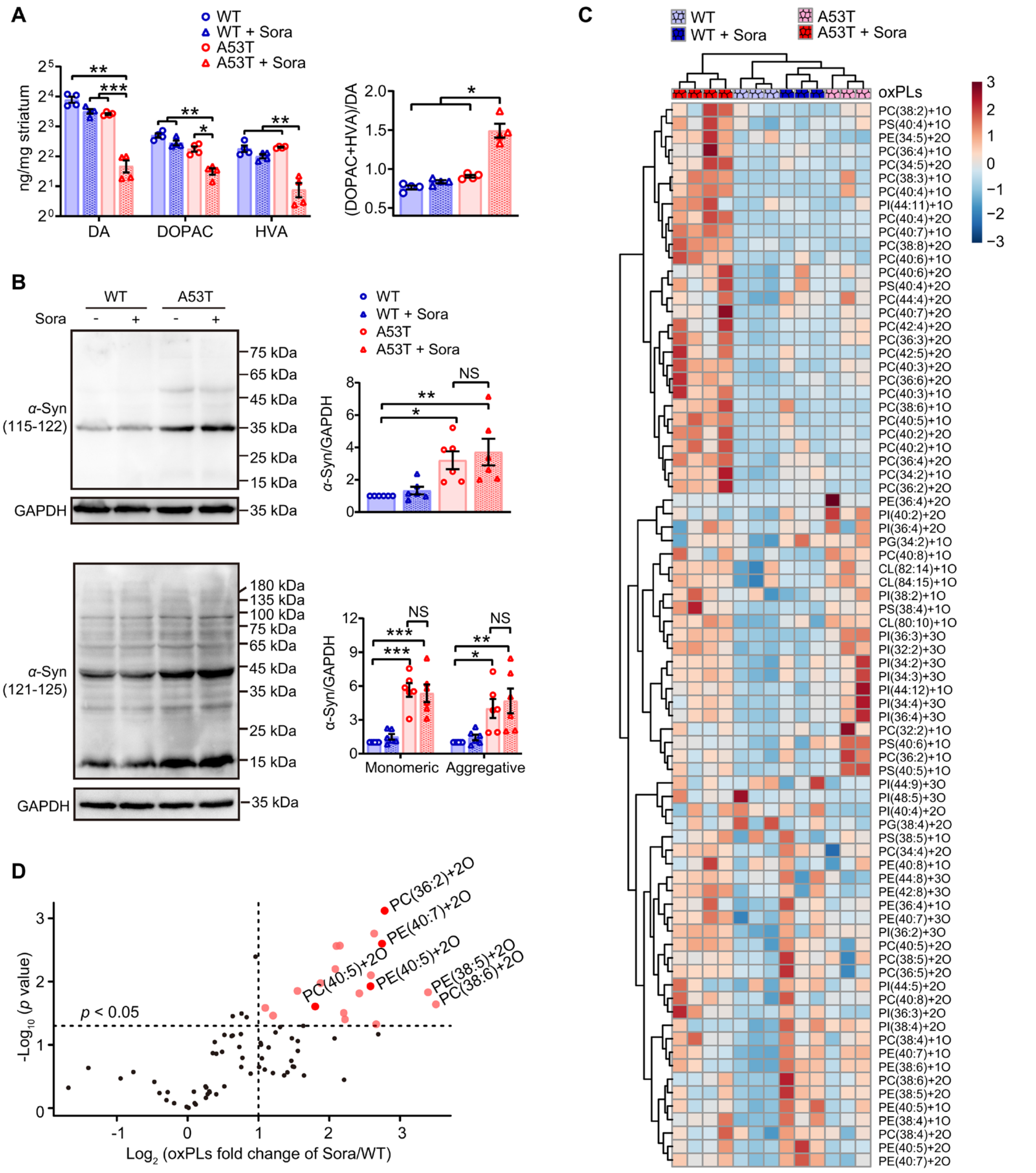

Figure S3. Phospholipid peroxidation occurred downstream of synucleinopathies. A53T transgenic mice were treated with low-dose sorafenib (Sora, 30 mg/kg as daily *i.p.*) for 2 weeks before measurements. (**A**) The contents of DA and the metabolites DOPAC and HVA in striatum were determined by LC-MS (left), and the turnover rate of DA and the metabolites

were calculated (right, $n = 4$ mice each group). $^{*}p < 0.05$, $^{**}p < 0.01$ and $^{***}p < 0.001$, by one-way ANOVA with Dunnett T3. (**B**) Western blotting and quantitative analysis of *α*-synuclein expression in midbrain ($n = 6$ mice each group). The antibodies (ab27766 and sc-7011-R) reacts with an epitope located in the region encoded by amino acids 115-122 and 121-125 of *α*-synuclein, respectively. $^{*}p < 0.05$, $^{**}p < 0.01$ and $^{***}p < 0.001$, by one-way ANOVA with LSD post-*hoc* test. NS, not significant. Data of oxidized phospholipids were extracted and displayed as (**C**) heatmap and (**D**) volcano plots showing the fold changes (X-axis) *vs* significance (Y-axis, by *t*-test). $n = 4$ mice in WT group, and 3 mice in the remaining three groups.

Supplementary figure 4

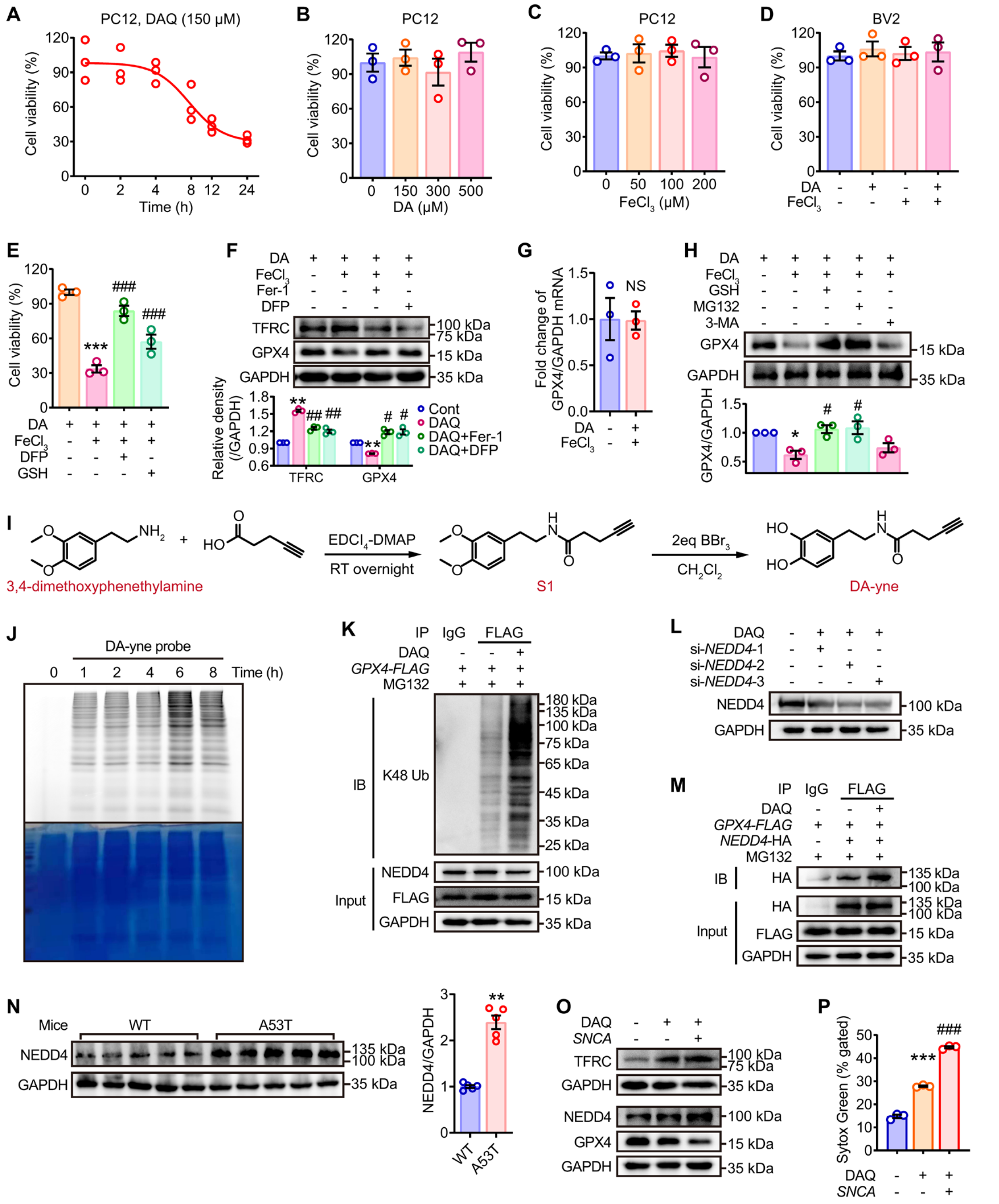

Figure S4. Dopamine and *α*-synuclein both contribute to NEDD4-mediated GPX4 ubiquitin proteasome degradation. (**A**) Cell viability was determined by MTT assay in PC12 cells treated with dopamine quinone (DAQ, 150 μM DA mixed with 150 μM $FeCl_3$) time-dependently. Cell viability was unaffected in PC12 cells treated with (**B**) DA or (**C**) $FeCl_3$ dose-dependently. (**D**) The viability

of BV-2 cells lacking DA-imported transporters was unaffected when treated with neither DA, $FeCl_3$, nor DAQ. (**E**) Cell viability was determined by MTT assay in PC12 cells treated with indicated agents. (**F**) Western blotting (upper) and quantitative analysis (bottom) of TFRC and GPX4 expressions in PC12 cells treated with indicated agents. (**G**) *Gpx4* gene expression was detected by quantitative real-time PCR assay. (**H**) Western blotting (upper) and quantitative analysis (bottom) of GPX4 expression in PC12 cells treated with indicated agents. DA, 150 μM. $FeCl_3$, 150 μM. DFP, deferiprone, 100 μM. GSH, glutathione, 100 μM. Fer-1, ferrostatin-1, 10 μM. MG132, 10 μM. 3-MA, 3-methyladenine, 10 mM. (**I**) Schematic diagram of the synthesis of alkyne-containing DA probe (DA-yne). (**J**) Time-dependent labeling profiles of DA-yne probe with PC12 cells, and 6-h treatment was chosen for the subsequent experiments. (**K**) The K48-specific ubiquitination level of GPX4 and the expression of NEDD4 were examined by Western blotting-based co-immunoprecipitation (co-IP) using PC12 cells transfected with *FLAG*-tagged *GPX4* plasmid. (**L**) The efficacy of *NEDD4* siRNAs were validated in HEK293 cells by Western blotting. (**M**) The interaction of GPX4 and NEDD4 was verified by Western blotting-based co-immunoprecipitation (co-IP) using HEK293 cells transfected with plasmids of *FLAG*-tagged *GPX4* and *HA*-tagged *NEDD4*. (**N**) Western blotting (left) and quantitative data (left) showed the NEDD4 upregulation in the midbrain of A53T mice (n = 5 mice each group). (**O**) Western blotting showed the upregulation of NEDD4 and TFRC, and the decline of GPX4 in DAQ-treated *SNCA*-overexpressed PC12 cells. (**P**) The cells with compromised membranes were penetrated by Sytox Green and determined by flow cytometry. All data represent mean ± SEM (for cells, n = 3 independent samples). $^{*}p < 0.05$, $^{**}p < 0.01$ and $^{***}p < 0.001$ *vs* the control group, $^{\#}p < 0.05$, $^{\#\#}p < 0.01$ and $^{\#\#\#}p < 0.001$ *vs* the DAQ-treated cells, by one-way ANOVA with Bonferroni (for **E**, **H**, **P**), Dunnett T3 (for **F**) tests or independent-samples t-test (for **G**, **N**). NS, not significant.

Supplementary figure 5

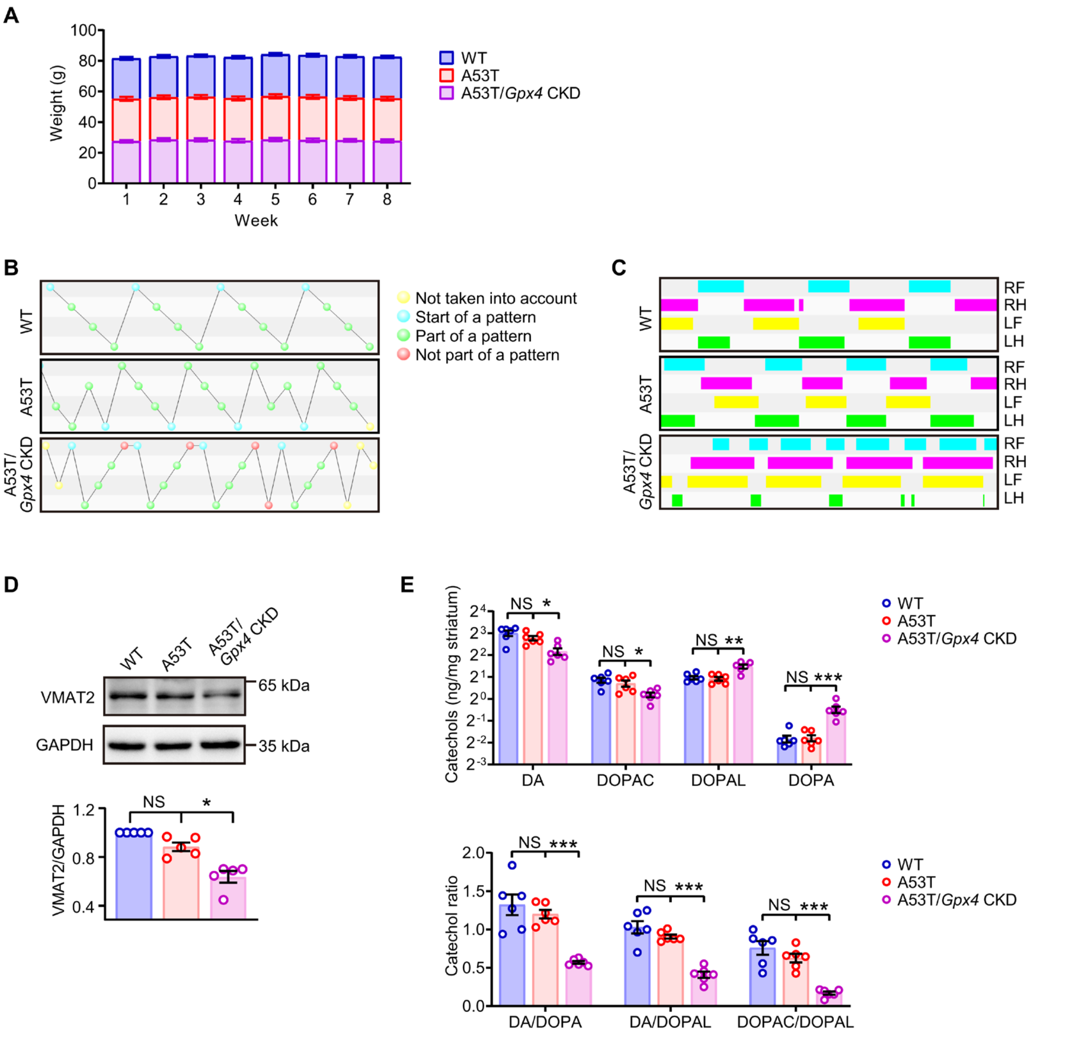

Figure S5. Conditional knockdown of *Gpx4* in substantia nigra accelerates the dysfunction of gait pattern in *SNCA*^{A53T}/*Gpx4*$^{+/flox}$ double transgenic mice. (**A**) Double hemizygous *SNCA*^{A53T}/*Gpx4*$^{+/flox}$ mice were obtained by cross-breeding *SNCA*^{A53T} and *Gpx4*$^{flox/flox}$, and were bilaterally injected adeno-associated virus (AAV)-*Cre* into the substantia nigra to acquire

a strain of A53T with conditional knockdown of *Gpx4* (CKD). No significant difference in the body weight of the mice of each strain was observed throughout the experiment. n = 10, 9, 8 mice in WT, A53T, A53T/*Gpx4* CKD, respectively. (**B-C**) The gait pattern was visualized as footfall pattern and footprint length. Green balls: normal pattern. Red balls: abnormal pattern. Blue balls: the beginning of a walking cycle. Yellow balls: data that were excluded from the test. RF, right front. RH, right hind. LF, left front. LH, left hind. (**D**) Western blotting (upper) and quantitative analysis (bottom) of VMAT2 expression in midbrain (n = 5 mice each group). (**E**) Levels of catechol, and catechol ratios in striatum tissue obtained from $SNCA^{A53T}/Gpx4^{+/flox}$ mice. (n = 6 mice each group). All data represent mean ± SEM. $^{*}p < 0.05$, $^{**}p < 0.01$ and $^{***}p < 0.001$, by one-way ANOVA with Dunnett T3 (for **D**) or Tukey HSD (for **E**) test. NS, not significant.

Supplementary figure 6

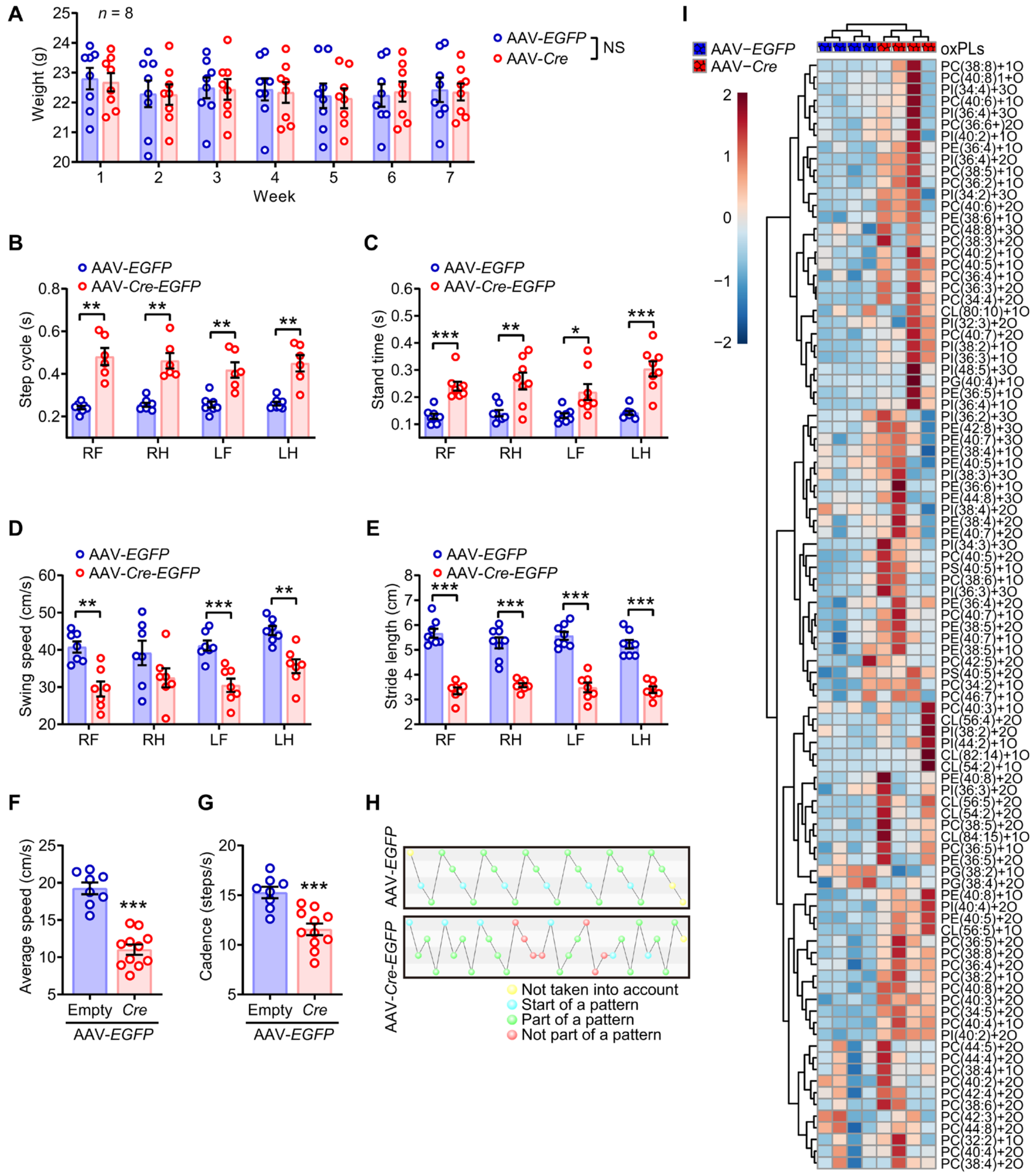

Figure S6. Conditional knockdown of *Gpx4* in substantia nigra leads to the disordered gait pattern and phospholipid oxidation in *Gpx4*$^{\text{flox/flox}}$ homozygous. (**A**) *Gpx4*$^{\text{flox/flox}}$ homozygous were bilaterally injected AAV-*Cre* into the substantia nigra which acquired a strain of conditional *Gpx4* knockdown mice. No significant difference in the body weight of the mice of each strain was

observed throughout the experiment (*n* = 8 mice each group). Disordered motor coordination of *Gpx4* CKD mice was assessed by Catwalk gait analysis including the (**B**) step cycle (*n* = 8 in *EGFP* group, *n* = 6 mice in *Cre* group), (**C**) stand time (*n* = 8 mice each group), (**D**) swing speed (*n* = 7 mice each group), (**E**) stride length (*n* = 8 in *EGFP* group, *n* = 7 mice in *Cre* group), (**F**) average speed (*n* = 8 in *EGFP* group, *n* = 11 mice in *Cre* group) and (**G**) cadence (*n* = 8 in *EGFP* group, *n* = 11 mice in *Cre* group). (**H**) The gait pattern was visualized as footfall pattern. RF, right front. RH, right hind. LF, left front. LH, left hind. Green balls: normal pattern. Red balls: abnormal pattern. Blue balls: the beginning of a walking cycle. Yellow balls: data that were excluded from the test. (**I**) Data of oxidized phospholipids were extracted and displayed as heatmap (*n* = 4 mice each group).

Supplementary figure 7

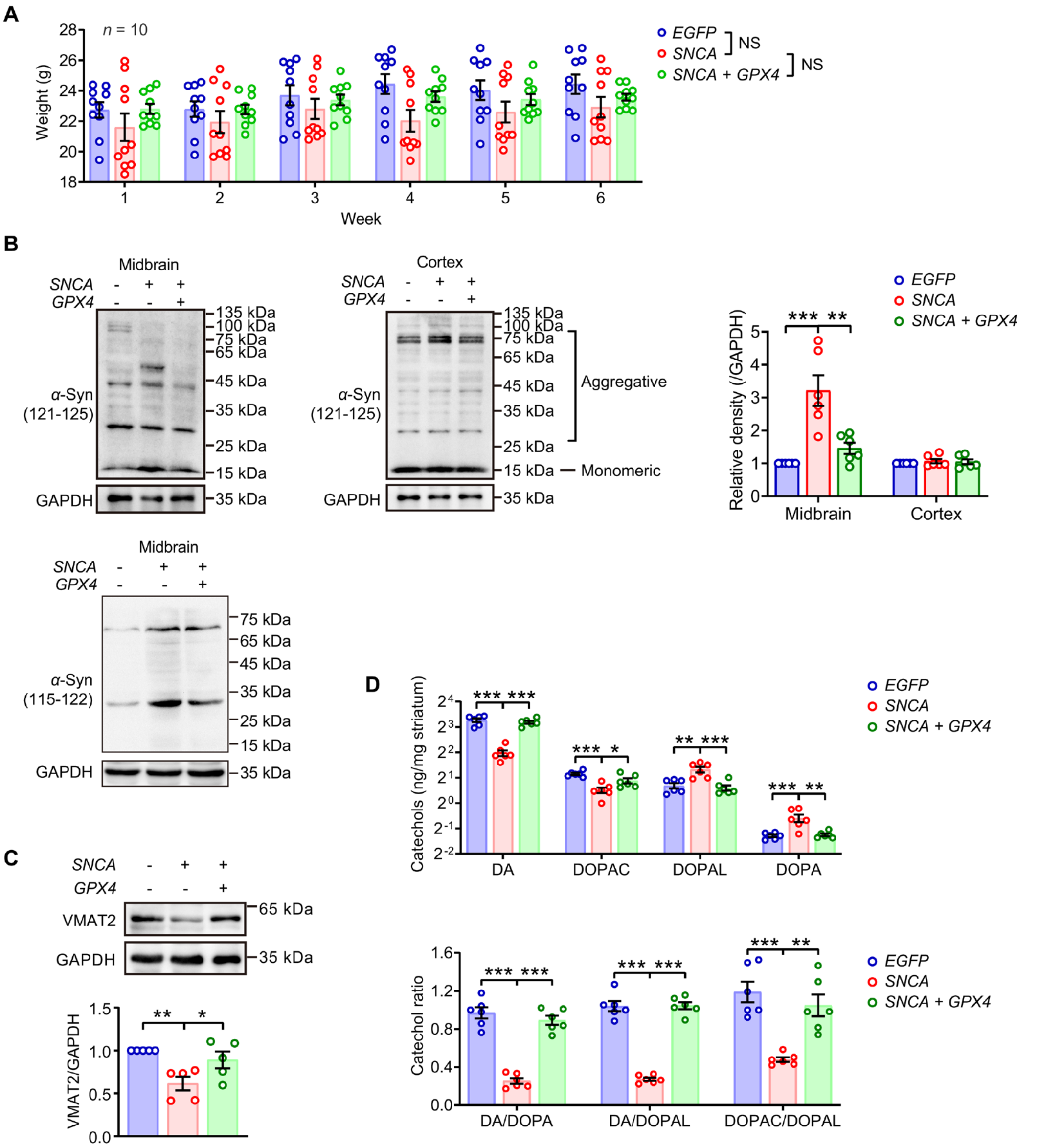

Figure S7. GPX4 replenishment alleviates synucleinopathy in mice overexpress *SNCA*. (**A**) WT mice were unilaterally injected AAV-loaded human *SNCA or GPX4* into the substantia nigra. No significant difference in the body weight of the mice of each strain was observed throughout the experiment (*n* = 10 mice each group). (**B**) *α*-Synuclein expression and aggregation was

reversed in midbrain, while unaffected in cortex, as quantified by Western blotting analysis (n = 6 mice each group). The antibodies (ab27766 and sc-7011-R) reacts with an epitope located in the region encoded by amino acids 115-122 and 121-125 of α-synuclein, respectively. $^{**}p < 0.01$ and $^{***}p < 0.001$, by one-way ANOVA with LSD post-*hoc* test. (**C**) Western blotting (upper) and quantitative analysis (bottom) of VMAT2 expression in midbrain (n = 5 mice each group). (**D**) Levels of catechol and catechol ratios in striatum tissue obtained from AAV-loaded *SNCA or GPX4* mice (n = 6 mice each group). All data represent mean ± SEM. $^{*}p < 0.05$, $^{**}p < 0.01$ and $^{***}p < 0.001$, by one-way ANOVA with Tukey HSD test. NS, not significant.

Supplementary methods

Pole test

Motor coordination was assessed by the time the mouse descends from the top of the pole to the landing of the two front paws. A 2 cm diameter ball was fixed on the top of an iron frame with a length of 50 cm and a diameter of 1 cm. The pole was covered with two layers of gauze to prevent it from slipping. Mice were trained 3 days ahead of the experiment. After training, the mouse was placed head-up on the ball-like shape, mice climbed to the bottom pole time was recorded, with a maximum observation time of 60 s. The test was repeated for 5 times to obtain the average value. To avoid interference, the experiment was carried out in a quiet environment.

Rotarod test

Mice were first trained to stay on the rod of the rotarod (Ugo Basile S.R.L., Gemonio VA, Italy) 3 days prior to the actual test and the speed was set to evenly accelerate from 5 rpm to 30 rpm in 300 s. After training, each mouse was repeatedly measured 5 times at the accelerated speed. Between each trial, there was an interval of 30 min break for the mice. The experiment was performed as follows: mice were placed on the rod and initially maintained at a constant speed of 5 r/min, allowing the mice to maintain a good balance on their respective runways for 15 s. The rod speed was increased from 5 r/min to 30 r/min within 300s until the mouse fell from the rod within the measurement time limit. The average on-rod time of three trials was reported. All tests and measurements were performed on the same day. Experiments were conducted in a quiet environment to avoid interference.

Gait analysis

To assess detailed functional changes in gait, gait analysis was performed using the CatWalk™ XT (Noldus Information Technology, Wageningen, The Netherlands), and the parameters were then calculated using the CatWalk XT software package. Three days before the test, the mice were trained to relieve stress. The experiment process is as follows: mice passed through the glass corridor, and the fluorescent lights illuminated the corridor, allowing the charge

coupled device (CCD) camera below to capture images of the footprint. Repeat the actual test until three consecutive uninterrupted runs were recorded. All tests and measurements were performed on the same day. Experiments were conducted in a quiet environment to avoid interference.

TH, GPX4 or 4-HNE staining of brain section

GTVision™ Anti-Mouse/Rabbit Universal Immunohistochemistry Kit (GenTech Biotechnology, Shanghai, China) was applied for observation of dopaminergic neurons. Mice were anesthetized with 3% isoflurane immediately after the behavior tests and were intracardially perfused with saline first, then with 4% paraformaldehyde in 0.1 M phosphate buffer (pH 7.4). Whole brains were immersed in 4% paraformaldehyde at 4 °C for 24 h, embedded with paraffin and cut into 5 μm-sections. After antigen retrieval, the sections were blocked in 10% 0.1 M normal goat serum and incubated with primary mouse antibodies overnight at 4 °C. After labeling with secondary antibody for 2 h at room temperature, the sections were stained with DAB and hematoxylin (for IHC), or with DAPI (for immunofluorescence), before observed under a microscope (for IHC, Olympus, Tokyo, Japan), or confocal microscope (for immunofluorescence, ZEISS, Germany). For IHC, the expression level of GPX4 and 4-HNE proteins was independently assessed by 2 pathologists who were blind to the data, based on the proportion and intensity of positive cells determined in 5 microscopic 40 × fields per mouse. Scores representing the proportion of positively stained were assigned as follows: 0, < 10%; 1, 11-25%; 2, 26-50%; 3, 51-75%; and 4, 76-100%. Next, an intensity score, which indicated the average intensity of positively stained cells，was assigned as follows: 0 (none), 1 (weak), 2 (intermediate), and 3 (strong) or 4 (significantly strong). The proportion and intensity scores were then multiplied to obtain a final score, which ranged 0 to 16.

AFADESI-MSI assay for determination of DA in mouse brain

AFADESI-MSI assay was conducted as described previously (1).The brains of mice were obtained after sacrifice, cut into 10 μm coronal sections at -20 °C on cryostat microtome, and the mounted onto microscope slides

(Thermo Fisher Scientific, Waltham, MA, USA). A set of adjacent tissue sections were fixed in acetone and then were stained with H&E for histological observation. Cryosections were dried in vacuum for 15 min before (±) MSI analysis in full MS (100-1000 m/z) scan mode.

LC-MS analysis for determination of GSH

Mice substantia nigra and striatum were collected on ice. Perchloric acid (0.1 M) was added according to the ratio of v/w = 10 μL:1 mg. The tissue was homogenized on ice for 4 min, centrifuged at 13,000 × *g* for 15 min in a refrigerated centrifuge. The supernatant was transferred and passed through a filter to draw a 20 μL sample for analysis. Analysis was conducted by LC-MS using a Triple Quad 4500 Mass Spectrometer (SCIEX). Analytes were separated on a C18 column (Acquity HSS T3, 1.8 μm, 2.1 mm × 100 mm, 40 °C, Waters) at a flow rate of 0.4 mL/min on an Exion LC AD system. Gradient: solvent A (water) and solvent B (acetonitrile), each containing 0.1% formic acid (v/v): 0-1.2 min isocratic of 2% B, 1.2-2.5 min linear gradient of 2 to 60% B, 2.5-4.5 min linear gradient of 60% to 95% B, 4.5-5.0 min isocratic of 95% B, and 5.0-6.5 min re-equilibration of 2% B. Positive and negative ion modes switching -4.5 kV; ion source temperature, 600 °C. Nebulizer gas and heater gas were set to 55 psi, and curtain gas was set to 30 abr. Data acquisition and processing were carried out using Analyst 1.6.2 software (SCIEX).

LC-MS analysis for determination of DA and metabolite

Mice substantia nigra and striatum were collected on ice. Perchloric acid (0.1 M) was added according to the ratio of v/w = 10 μL:1 mg. The tissue was homogenized on ice for 4 min, centrifuged at 13,000 × *g* for 15 min in a refrigerated centrifuge. The supernatant was transferred and passed through a filter to draw a 40 μL sample for analysis. Analysis was conducted by LC-MS using a Dionex UltiMate 3000 DGLC standard system (Thermo Fisher Scientific). Analytes were separated on a C18 column (Acquity HSS T3, 1.8 μm, 2.1 mm × 100 mm, 40 °C, Waters) at a flow rate of 0.3 mL/min on an Dionex UltiMate 3000 UHPLC system. Gradient: solvent A (water) and solvent B (acetonitrile), each containing 0.1% formic acid (v/v): 0-1 min isocratic of 2% B,

1-2 min linear gradient of 2 to 60% B, 2-3.5 min linear gradient of 60% to 95% B, 3.5-4.5 min isocratic of 95% B, and 4.5-10.0 min re-equilibration of 0% B. Positive ion modes was +3.0 kV and negative ion modes was -2.8 kV. Capillary temperature was 350 °C. The S-lens Rf level was 65, and curtain gas was set to 30 arb. Data acquisition and processing were carried out using Analyst 1.6.2 software (SCIEX).

MTT assay

Cells were dispensed in 96-well plate at a density of 1 × 10^5 cells per well. After 24 h incubation, cells were treated with the tested reagents for the indicated periods of time and stained with MTT. Optical density was measured using an ELISA reader (Thermo Fisher Scientific, Waltham, MA, USA).

LDH assay

Tests were conducted according to the manufacturer's instructions (Nanjing Jiancheng Bioengineering Institute, Nanjing, China). Optical density was measured using an ELISA reader (Thermo Fisher Scientific).

Liperfluo assay

Cells were dispensed in dishes at a density of 5 × 10^5 cells. After treatment, cells were incubated with Liperfluo (DojinDo, Kumamoto, Japan) for 30 min at 37 °C. Then the cells were collected for analysis in a BD FACSCanto II flow cytometer. The excitation and emission wavelengths are 524 nm and 535 nm, respectively.

Iron staining

Cells were dispensed in dishes at a density of 5 × 10^5 cells. After treatment, cells were incubated with FerroOrange (DojinDo) for 30 min at 37 °C. Then the cells were imaged using a ZEISS LSM 800 confocal laser scanning microscope (Carl Zeiss, Oberkochen, Germany).

LC-MS/MS-based phospholipidomics analysis

Phospholipids were separated by a Dionex UltiMate 3000 DGLC standard

system (Thermo Fisher Scientific) at a flow rate of 0.2 mL/min on normal-phase column (Phenomenex Luna Silica, 3 μm, 150 × 2.0 mm, Danaher Corporation, Washington DC, USA). The column temperature was maintained at 35 °C. The mobile phase consisted of 10 mM ammonium formate in propanol/hexane/water (285:215:5, v/v/v, solvent A) and 10 mM ammonium formate in propanol/hexane/water (285:215:40, v/v/v, solvent B). The linear gradient conditions were as follows: 0 min, 10% B; 20 min, 32% B; 30 min, 70% B; 32 min, 100% B; 58 min, 100% B; 60 min, 10% B; 75 min, 10% B. The injection volume was 2 μL.

MS/MS analysis of phospholipids was performed on a Q-Exactive Hybrid Quadrupole-Orbitrap mass spectrometer (Thermo Fisher Scientific). Analysis was performed in full MS negative mode at resolution setting of 70,000 and data-dependent-MS/MS mode at resolution setting of 17,500. For MS, the scan range was 400-1,800 m/z and the maximum ion injection time was 600 ms using 1 microscan per MS scan. For MS/MS, high energy collision induced dissociation (HCD) analysis was performed with the collision energy set to 24 eV and the maximum ion injection time of 200 ms. The inclusion list included all species of phospholipids and their oxidized/deuterated products. An isolation window of 1.0 Da was set for the MS and MS/MS scans. Capillary spray voltage was 3.0 kV, and capillary temperature was 320 °C. The S-lens Rf level was 60.

In Situ **and** ***in vitro*** **proteome labeling**

Cells were dispensed in dishes at a density of 5×10^5 cells. After treatment, cells were incubated with 2 mL probe-containing fresh medium for 6 h. Then the cells were treated with RIPA lysis and extraction buffer containing 2.5% Chaps and phosphatase inhibitor, and then lysed with sonication on ice. The protein concentrations were determined by using the BCA protein assay and diluted to 1 mg/mL with PBS. A freshly pre-mixed click chemistry reaction cocktail was added (50 μM TAMRA-N_3 from 25 mM stock solution in DMSO, 100 μM TBTA from 50 mM stock solution in DMSO, 1 mM TCEP from 1 M stock solution in deionized water, and 1 mM $CuSO_4$ from 1 M stock solution in deionized water. All the stock solutions above were freshly prepared). The reaction was further incubated for 2 h prior to addition of pre-chilled acetone (-

20 °C). The precipitated proteins were subsequently collected by centrifugation (14,000 × *g* for 10 min at 4 °C) and washed twice with 1 mL of prechilled acetone. The samples were dissolved in 1 × SDS loading buffer and heated for 15 min at 95 °C. The proteins were separated by a 10% SDS-PAGE gel and then visualized by in-gel fluorescence scanning (Typhoon FLA 9500, GE Healthcare, Chicago, IL, USA).

Lists of resources

Table 1. List of agents

Category	Company	Product No.
Chemicals		
RSL3	Selleckchem	S8155
Ferrostatin-1 (Fer-1)	Selleckchem	S7243
Erastin	Selleckchem	S7242
Doxycycline hydrochloride	Sigma	D9891-1G
Sorafenib	Meilunbio	MB1666
MTT	Sigma	M2128-5G
DA standard	Sigma	H8502
Cycloheximide (CHX)	Selleck	S7418-200 mg
MG132	MedChemExpress	HY-13259-25 mg
Trifluoroacetic acid (TFA)	ACMEC	T74920-500 mL
Urea	Macklin	U820349
Azide-PEG3-biotin conjugate	TCI	A2523-100mg
NeutrAvidin agarose	Thermo Fisher Scientific	29202
TCEP	Macklin	T917415
TBTA	RHAWN	R055315
BCA color developing fluid	Thermo Fisher Scientific	SLZ60212
Fluorogenic dyes		
Liperfluo	Dojindo	L248
C11 Bodipy	Invitrogen	D3861
SYTOX Green	Thermo Fisher Scientific	S7020
FerroOrange	DOJINDO	F374
DAPI	Beyotime	C1002
Critical Commercial kits		
Iron Assay Kit	Abcam	ab83366
DAB color development kit	Gene Tech	GK347010
MDA assay kit	Beyotime	S0131
Reverse transcription kit	TransGen	AT311-03
SYBR Green qPCR Mix	TransGen	AQ141-01
Hematoxylin and eosin staining kit	Beyotime	C0105
LDH cytotoxicity assay kit	Beyotime Biotechnology	C0017
Antibodies		

Rabbit anti-GPX4	Abcam	ab125066
Rabbit anti-4-HNE	Abcam	ab46545
Rabbit anti-*α*-synuclein	Abcam	Ab27766
Rabbit anti-*α*-synuclein	Santa Cruz Biotechnology	SC-7011-R
Rabbit anti-TH	Abcam	ab112
Rabbit anti-TFRC (CD71)	Proteintech	10084-2-AP
Rabbit anti-NEDD4	Bioss	bs-7877R
Rabbit anti-Ubiquitin	CST	3933S
Rabbit anti-VMAT2	Abcam	ab259970
Mouse anti-FLAG	Proteintech	66008-3-IG
Rabbit anti-HA	Proteintech	51064-2-AP
Mouse anti-GAPDH	FuDe	FD0063
Goat Anti-Rabbit IgG HCS	Abkine	A25222-1
Goat anti mouse-HRP	FuDe	FDM007
Goat anti rabbit-HRP	FuDe	FDR007
Others		
TritonX-100	Sigma	900502
PBCAG-eGFP	Addgene	40973
RIPA lysis buffer	Beyotime	P0013C
Trizol	Invitrogen	15596018
DMEM basal medium	Gibco	C11995500BT
Horse serum (HS)	Gibco	26050088
Fetal bovine serum (FBS)	PAN	P30-3302

Table 2. List of AAVs, siRNAs and plasmids

Category	Company
rAAV-hSyn-CRE-EGFP-WPRE-hGH polyA (MK854762.1)	BrainVTA (Wuhan, China)
AAV-hsyn-hGPX4-3flag-P2A-EGFP-WPRE-PA (NM_002085.5)	BrainVTA (Wuhan, China)
AAV-SNCA (NM_001146054)	Taitool Bioscience (Shanghai, China)
si-NEDD4	RiboBio
pcDNA3.1-NEDD4-HA	Transheep (Shanghai, China)
pcDNA3.1-GPX4-FLAG	BrainVTA (Wuhan, China)
pCMV-Ub-myc	Transheep (Shanghai, China)

Table 3. List of siRNAs of *NEDD4*

siRNA No.	Target Sequence
si-r-*NEDD4*-1	CCAAGAATGGAGAGACCAT
si-r-*NEDD4*-2	GCTCGCAAACCTGTATCTT
si-r-*NEDD4*-3	CCAGAGCACATACCTGCTT

Table 4. List of primer sequences used in RT-qPCR

Species	Primer name	Sequence (5'→3')
Rat	*Gpx4*-F	ATAAGAACGGCTGCGTGGTGAAG
	Gpx4-R	TAGAGATAGCACGGCAGGTCCTTC
Rat	*Pla2g6*-F	GCCGACTACCACTTCCCATTCATAC
	Pla2g6-R	GCTGATCGTTGGAGGCTGAGTTC
Rat	*Slc7a11*-F	CCATCATCATCGGCACCGTCATC
	Slc7a11-R	TACTCCACAGGCAGACCAGAACAC
Rat	*Alox15*-F	AGCTGTGCAAGACGACTATGAACTG
	Alox15-R	CGGGACTGAAGAGAGGTAGGGAAG
Rat	*Alox5*-F	ACCTATTCCTCCCTGTGCTTCCC
	Alox5-R	CCACACGAGCAGTCCATCATCAC
Rat	*Tfrc*-F	GTTCCCCGTTGTTGAGGCAGAC
	Tfrc-R	GATGACTGAGATGGCGGAAACTGAG
Rat	*Gapdh*-F	AGTTCAACGGCACAGTCAAGGC
	Gapdh-R	CGACATACTCAGCACCAGCATCAC
Mus	*Gpx4*-F	ATAAGAACGGCTGCGTGGTGAAG
	Gpx4-R	TAGAGATAGCACGGCAGGTCCTTC
Mus	*Pla2g6*-F	CGGCCTGAACCAGGTAAACAA
	Pla2g6-R	GTTGCAGCGGGCATTACAG
Mus	*Slc7a11*-F	CTATTTTACCACCATCAGTGCG
	Slc7a11-R	ATCGGGACTGCTAATGAGAATT
Mus	*Ptgs2*-F	TGCACTATGGTTACAAAAGCTGG
	Ptgs2-R	TCAGGAAGCTCCTTATTTCCCTT

Mus	*Tfrc*-F	TCACACTCTCTCAGCTTTAGTG
	Tfrc-R	TGGTTTCTGAAGAGGGTTTCAT
Mus	*Ncoa4*-F	ACCAGCCTAGAGGTGTGGAGATTG
	Ncoa4-R	GTCCTGATGGTTCTGGGCAAGC
Mus	*Alox15*-F	GGCTCCAACAACGAGGTCTAC
	Alox15-R	CCCAAGGTATTCTGACACATCC
Mus	*Alox12*-F	TCCCTCAACCTAGTGCGTTTG
	Alox12-R	CCTCGGGAACGTCGAAGTC
Mus	*Alox5*-F	ACTACATCTACCTCAGCCTCATT
	Alox5-R	GGTGACATCGTAGGAGTCCAC
Mus	*Acsl4*-F	CAATAGAGCAGAGTACCCTGAG
	Acsl4-R	TAGAACCACTGGTGTACATGAC
Mus	*Lpcat3*-F	CTACCCGTTGGCTCTGTTTTAC
	Lpcat3-R	TGAAGCACGACACATAGCAAG
Mus	*Dmt1*-F	TACCTAGACCCAGGAAACATCG
	Dmt1-R	CACTCCAAGTCTCGCTGCAA
Mus	*Gapdh*-F	AAGAAGGTGGTGAAGCAGG
	Gapdh-R	GAAGGTGGAAGAGTGGGAGT

Author contributions

JS conducted most of the in vitro experiments and acquired and analyzed the core data, including BLI kinetic experiments, synthesis of DA probes, modification site analysis of DAQ to GPX4 protein, proteomics for E3 ligase, and most of the LC-MS/MS-based phospholipidomics, and thus listed as the first co-author. XML conducted most of the in vivo experiments and acquired and analyzed data, including interventions in transgenic mice by virus injection, behavioral tests, and part of phospholipidomics, and thus listed as the second co-author. DHL conducted most of the in vivo experiments and acquired and analyzed data, including behavioral tests and part of phospholipidomics, and thus listed as the third co-author. MW assisted in conducting in vivo experiments and acquiring data. KL assisted in conducting in vitro experiments and acquiring data. SRL assisted in conducting modification site analysis of DAQ to GPX4 protein. ZQL supervised and assisted in modification site analysis of DAQ to GPX4 protein. CJZ conducted the preparation of GPX4 recombinant. ZMZ supervised and conducted the preparation of GPX4 recombinant. CYY assisted in conducting in vivo experiments and acquiring data. MHP assisted in conducting in vivo experiments and acquiring data. HBG conducted the LC-MS/MS-based experiments and acquired data. JCF conducted the LC-MS/MS-based experiments and acquired data. YFC supported the LC-MS platform and consulted regarding the data analysis. FH supervised and advised the project. WYS conducted the LC-MS/MS-based experiments and analyzed data. HK supervised and advised the project. YFL advised the project. WJD designed and advised the project, analyzed and approved data, prepared all the figures and tables, and wrote the manuscript. GLJ supervised and advised the project. LZ advised the project and revised the manuscript. RRH designed and supervised the project, revised and approved the manuscript.

Reference

1. He J, Sun C, Li T, Luo Z, Huang L, Song X, et al. A Sensitive and Wide Coverage Ambient Mass Spectrometry Imaging Method for Functional Metabolites Based Molecular Histology. *Adv Sci (Weinh).* 2018;5(11):1800250.
